# The landscape of cancer-associated fibroblasts in colorectal cancer liver metastases

**DOI:** 10.7150/thno.72853

**Published:** 2022-10-31

**Authors:** Ambre Giguelay, Evgenia Turtoi, Lakhdar Khelaf, Guillaume Tosato, Ikrame Dadi, Tommy Chastel, Marie-Alix Poul, Marine Pratlong, Stefan Nicolescu, Dany Severac, Antoine Adenis, Olivia Sgarbura, Sébastien Carrère, Philippe Rouanet, François Quenet, Marc Ychou, Didier Pourquier, Pierre-Emmanuel Colombo, Andrei Turtoi, Jacques Colinge

**Affiliations:** 1 Institut régional du Cancer de Montpellier (ICM)-Val d'Aurelle, Montpellier, France.; 2 Université de Montpellier, Montpellier, France.; 3 Cancer Bioinformatics and Systems Biology Team, INSERM U1194, Montpellier, France.; 4 Tumor Microenvironment and Resistance to Treatment Lab, INSERM U1194, Montpellier, France.; 5 Department of Pathology, Institut régional du Cancer de Montpellier (ICM)-Val d'Aurelle, Montpellier, France.; 6 Biocampus, CNRS, INSERM, Université de Montpellier, Montpellier, France.; 7 Department of Medical Oncology, Institut régional du Cancer de Montpellier (ICM)-Val d'Aurelle, Montpellier, France.; 8 Department of Surgery, Institut régional du Cancer de Montpellier (ICM)-Val d'Aurelle, Montpellier, France.; 9 Gunma University Initiative for Advanced Research (GIAR), Maebashi, Gunma, Japan.

**Keywords:** single-cell, tumor microenvironment, CAF, liver metastasis, LTBP2.

## Abstract

**Rationale:** Patients with colorectal cancer die mainly due to liver metastases (CRC-LM). Although the tumor microenvironment (TME) plays an important role in tumor development and therapeutic response, our understanding of the individual TME components, especially cancer-associated fibroblasts (CAFs), remains limited.

**Methods:** We analyzed CRC-LM CAFs and cancer cells by single-cell transcriptomics and used bioinformatics for data analysis and integration with related available single-cell and bulk transcriptomic datasets. We validated key findings by RT-qPCR, western blotting, and immunofluorescence.

**Results:** By single-cell transcriptomic analysis of 4,397 CAFs from six CRC-LM samples, we identified two main CAF populations, contractile CAFs and extracellular matrix (ECM)-remodeling/pro-angiogenic CAFs, and four subpopulations with distinct phenotypes. We found that ECM-remodeling/pro-angiogenic CAFs derive from portal resident fibroblasts. They associate with areas of strong desmoplastic reaction and Wnt signaling in low-proliferating tumor cells engulfed in a stiff extracellular matrix. By integrating public single-cell primary liver tumor data, we propose a model to explain how different liver malignancies recruit CAFs of different origins to this organ. Lastly, we found that LTBP2 plays an important role in modulating collagen biosynthesis, ECM organization, and adhesion pathways. We developed fully human antibodies against LTBP2 that depleted LTBP2+ CAFs *in vitro*.

**Conclusion:** This study complements recent reports on CRC-LM CAF heterogeneity at the single-cell resolution. The number of sequenced CAFs was more than one order of magnitude larger compared to existing data. LTBP2 targeting by antibodies might create opportunities to deplete ECM-remodeling CAFs in CRC-LMs. This might be combined with other therapies, e.g., anti-angiogenic compounds as already done in CRC. Moreover, we showed that in intrahepatic cholangiocarcinoma, in which ECM-remodeling CAF proportion is similar to that of CRC-LM, several genes expressed by ECM-remodeling CAFs, such as *LTBP2*, were associated with survival.

## Introduction

Metastases account for >90% of cancer-related deaths worldwide. Liver is a dissemination hub for the deadliest malignancies, including colorectal, breast, lung and pancreatic cancer. More than 50% of patients with advanced colorectal cancer (CRC) develop liver metastases (CRC-LM) within five years after the primary tumor resection. Only one third of these patients are operable, and the others are managed with systemic chemotherapy 1, 2 with or without targeted therapy. In most cases, this leads to tumor resistance and progression. Consequently, the survival of patients with CRC-LM rarely exceeds five years.

The tumor microenvironment (TME) of solid tumors offers promising opportunities for cancer treatment 3. The success of immune checkpoint inhibitors (ICIs), *e.g.*, PD-L1 blockade, demonstrates that disruption of the cellular crosstalk between cancer cells and the TME can lead to therapeutic success. However, only a minority of patients benefit from ICIs, especially in CRC 4, indicating that more studies are needed. Cancer-associated fibroblasts (CAFs) are among the most abundant and versatile components of the tumor stroma. They are implicated in all the hallmarks of cancer 5, and growing evidence suggests that CAFs display tumor suppressive and also tumor promoting functions 6. Recent single-cell studies in primary breast tumors strengthened the long-held suspicion that CAFs do not have a unique phenotype within a single tumor 7,8. Some CAF populations may display immunosuppressive properties, but not others. In addition to their phenotypic heterogeneity, CAFs can originate from multiple sources besides tissue-resident fibroblasts 9. In liver, there are two obvious physiological sources: resident portal fibroblasts (PFs) and hepatic stellate cells (HSCs) 10. PFs reside in portal spaces where they produce the connective tissue containing the bile duct, portal vein and artery (three essential liver structures). HSCs are vitamin A-storing and lipid droplet-containing cells that are found in the Disse space. A considerable body of data shows that HSCs can be activated upon liver injury, either through secreted factors or by immune cells directly, including liver resident macrophages (Kupffer cells) 11.

A first single-cell atlas of human healthy and cirrhotic liver 12 showed that there are at least four populations of mesenchymal cells in liver. Three of these populations show different degrees of association with the response to liver injury: HSCs that strongly express *RGS5*, cells that strongly express collagens and *PDGFRA* but lack *RGS5* expression, and a population of vascular smooth muscle cells (VSMCs) that strongly express *MYH11*. RGS5-expressing pericytes and VSMCs have been identified also among lung tumor CAFs in other single-cell studies 13 and in various primary tumor types 14. Two recent reports established the first single-cell atlases of CRC-LM composition (including CAFs), but their focused on tumor composition changes upon chemotherapy 15 or on a pan-population description with limited analysis of CAF heterogeneity 16. In the present study, we sequenced one order magnitude more CAFs and phenotypically described a CAF subset that is mostly responsible for the desmoplastic reaction in CRC-LM. We used CRC-LM samples from a cohort of patients who received chemotherapy before surgery, which is the standard of care for CRC-LM in France and a common procedure worldwide 17.

## Results

### CRC-LM CAFs comprise distinct subpopulations

**Figure 1:**
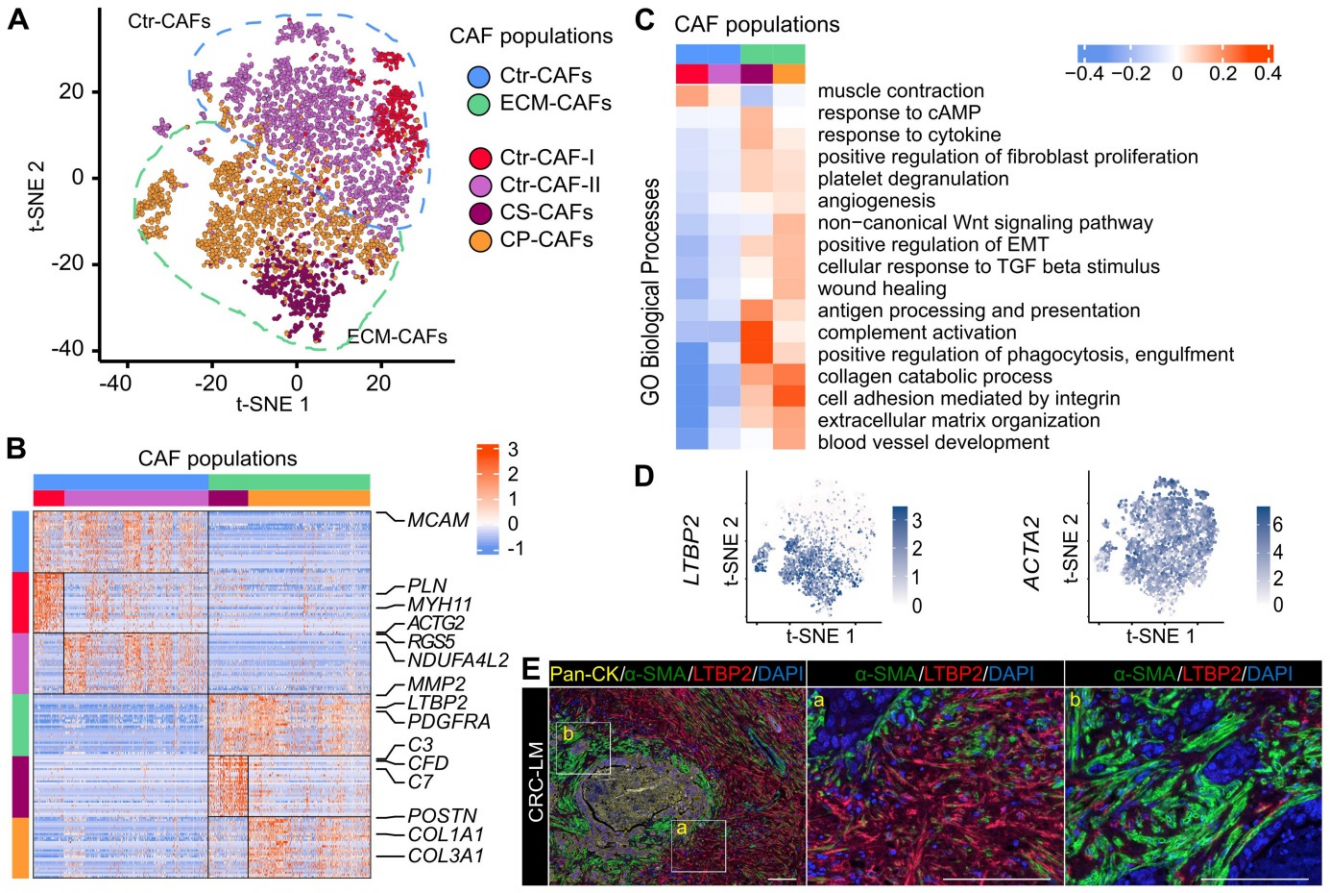
CAF heterogeneity. **A.** Analysis of the individual transcriptomes of 4,397 CAFs identified two main populations (ECM- and Ctr-CAFs) and four more specialized populations. **B.** Expression of the 30-gene signatures for each CAF population and subpopulation. **C.** Biological processes showing the different activity levels in the CAF populations. **D.** *LTBP2* and *ACTA2* expression in CAFs. **E.** Protein expression of Pan-CK (cancer cells), α-SMA (all CAFs) and LTBP2 (ECM-CAFs) in CRC-LM. Representative image of nine CRC-LM samples; scale bars,100 μm. Nuclei were counterstained with DAPI.

In this study, we isolated CAFs from six liver metastases resected from five patients, aged between 62 and 83 years (average 74 years). All patients received chemotherapy before surgery (Table S1). We purified CAFs using a triple negative (TN) selection strategy and flow cytometry (EPCAM-/CD45-/CD31-/LiveDead-) because of the absence of universal CAF-surface markers and the potential heterogeneity of this cell population. Other recent single-cell CAF studies used a similar procedure 7,8. After data quality filtering and elimination of few contaminating cells (n=215, mainly hepatocytes, Figure S1), we obtained the individual transcriptomes of 4,397 CAFs (Table S2). Using the 1,500 most variable genes for hierarchical clustering (Materials and Methods), we could cluster CAFs into two major populations that then clustered in two more specific subpopulations (Figure 1A). Then, we used the top 30 most significant genes found by differential gene expression analysis between CAF populations and subpopulations to define gene signatures for each population and subpopulation (Figures 1B, S2, and Table S3).

All CAF populations harbored genes linked to typical CAF functions, such as genes encoding collagen and ECM-related components (Figure S3). However, the activity levels of several biological processes varied among these populations (Figure 1C for an overview and Figures S4-S9 for more details). One of the two major populations featured genes involved in ECM remodeling (*e.g.*, *MMP2* expression in Figure S10) and collagen production (Figure S3). Therefore, we named this population ECM-remodeling CAFs (ECM-CAFs). *FAP,* a marker of fibroblast activation and proliferation, and *PDGFRA*, a marker of connective tissue remodeling 18, were also specifically expressed by ECM-CAFs (Figure S10). The second major CAF population expressed blood vessel wall markers, such as *RGS5* and *MCAM*. This population also strongly expressed *MYH11*, a contractility marker. Accordingly, we called the second major CAF population contractile CAFs (Ctr-CAFs).

*ACTA2* was expressed by all CAFs. Conversely, *LTBP2*, which was part of the ECM-CAF signature, was expressed only in this population (Figure 1D). Similarly, in an independent dataset of six patients with CRC-LM 15, we found that *ACTA2* was specifically expressed by all CAFs and *LTBP2* by a CAF subpopulation (Figure S11). Therefore, we used these two proteins as *bona fide* markers for these populations. In CRC-LM tissue, LTBP2/α-SMA double staining revealed distinct areas: some contained α-SMA+ CAFs and LTBP2-expressing cells (Figure 1E, zone marked “a”), while other areas contained mainly α-SMA+ CAFs (Figure 1E, zone marked “b”). LTBP2-expressing cells were specifically localized in the vicinity of α-SMA+ cells. Protein co-localization could not be observed because α-SMA is a cytoskeletal protein and LTBP2 is mainly secreted. For the sake of simplicity, we only distinguished LTBP2+ and α-SMA+ CAFs in the subsequent analyses, and we assumed that LTBP2+ CAFs always expressed α-SMA, as shown in two independent single-cell datasets and Figure 1E.

Transcriptomic data allowed further decomposing the two main CAF populations. We identified an ECM-CAF subpopulation that we called collagen-producing CAFs (CP-CAFs). CP-CAFs, which represented the majority of ECM-CAFs, were even more active in many biological processes (Figure 1C). These included collagen production (Figure S3), TGF-β response (as illustrated by *POSTN* and *INHBA* expression; Figure S10), angiogenesis (*VEGFC* and *UNC5B* and *SRPX2*, two angiogenesis-involved genes 19, 20; Figure S10), and Wnt signaling. The second ECM-CAF subpopulation expressed complement genes (*e.g.*,* C3*, *C7* and *CFD*; Figure S10), and also *CLU* at an intermediary level (Figure S10). Complement genes have a potential immunosuppressive effect in some tumors, including CRC, particularly when regulators of the complement cascade, such as *CLU,* are co-expressed 21. We called these CAFs complement-secreting CAFs (CS-CAFs). Similarly, we could classify Ctr-CAFs into two subpopulations. The first subpopulation (Ctr-CAFs of type I, Ctr-CAF-I) expressed additional contractility markers, such as *PLN* and *ACTG2* (Figures 1C and S10); however, this was not an exclusive feature of this subpopulation. They expressed *CLU* at a higher level than CS-CAFs (Figure S10). The second Ctr-CAF subpopulation (Ctr-CAFs of type II, Ctr-CAF-II) displayed an average CAF phenotype (Figures 1C and S3).

A recent report on TME heterogeneity in different cancer types 14 identified five commonly found CAF populations. We matched our gene signatures with these data, and found highly significant overlaps (Table S4). We also obtained significant overlap of the ECM-CAF gene signature with a CAF population named CAF-S1 that is enriched in triple-negative breast tumors 7 (Table S5). Moreover, we checked the expression of our different CAF gene signatures in the CRC-LM single-cell dataset published by Che *et al.* 15. This dataset includes the transcriptomes of 258 CAFs that were obtained from six patients. The expression levels of our signature genes in these 258 CAFs reproduced the clusters we observed in our data (Figures S12 and S13A). In our data, each metastasis sample harbored all CAF populations, with the exception of the P3_MP sample that was devoid of Ctr-CAF-I although 622 CAFs from this tumor were sequenced (Table S2). This suggests inter-patient heterogeneity. All our patients received chemotherapy before surgery, while the dataset by Che *et al.* included treatment-naive and treated CRC-LM specimens. However, we did not find any significant compositional difference (Figure S13B).

### Origin of CAF populations

**Figure 2:**
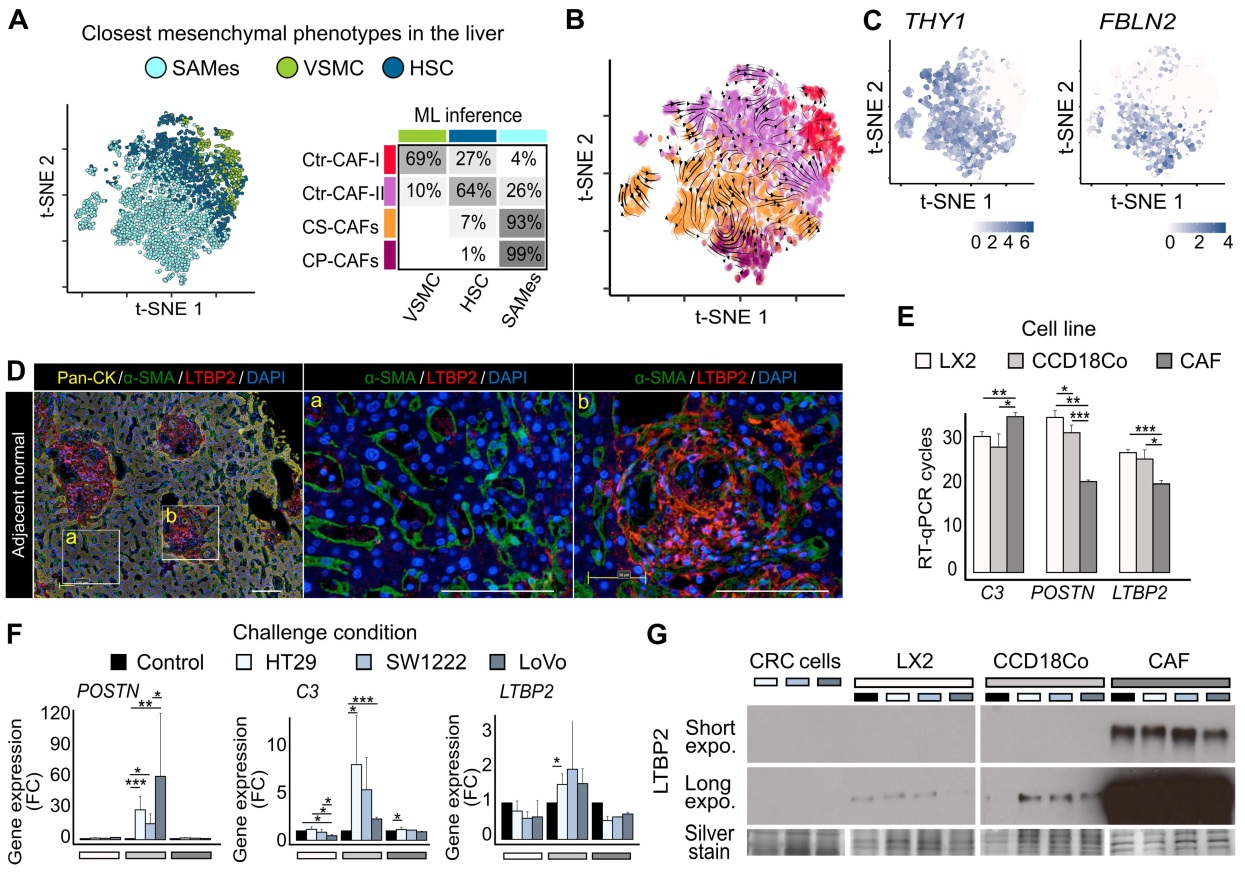
CAF origin. **A.** Machine learning (ML) prediction of CAF origin using mesenchymal cell transcriptome data from healthy and cirrhotic human livers (the table on the right shows the rate of closest mesenchymal phenotype assignment to each CAF population.) **B.** RNA velocity analysis indicating a seamless flow between ECM-CAF subtypes, in agreement with a common SAMes origin. Conversely, transitions between ECM- and Ctr-CAFs, and Ctr-CAF-II and Ctr-CAF-II seem more random, in agreement with their distinct origins. **C.** Portal fibroblast genes not expressed by HSCs strongly associate with ECM-CAFs. **D.** Multiplexed immunofluorescence staining of Pan-CK (hepatocytes), α-SMA (HSCs), and LTBP2. The presence of LTBP2+ CAFs in CRC-LM samples is limited to portal regions of the normal adjacent liver tissue. LM, liver metastasis; D, desmoplasia; and NL, normal liver. Representative images of nine CRC-LM samples; scale bars, 100μm. Nuclei were counterstained with DAPI. **E-F.** RT-qPCR analysis of *C3*, *POSTN*, and *LTBP2* expression in LX2 and CCD18Co cells, and CRC-LM-CAFs incubated with CM from CRC cell lines (HT29, SW1222, and LoVo). Basal levels (**E**) and gene fold-change relative to control (**F**). **G.** Western blot analysis of LTBP2 abundance in conditioned medium samples. Panels E and F: *P < 0.05, **P < 0.01, ***P < 0.001.

To attempt understanding CRC-LM CAF phenotypic heterogeneity, we compared our data with an atlas of healthy and cirrhotic liver cell types 12. In this liver atlas, three populations of relevant mesenchymal cells are identified: VSMCs, HSCs, and cells strongly associated with fibrosis that express *PDGFRA*, but not *RGS5* and were called scar-associated mesenchymal cells (SAMes). We repeated our procedure to obtain gene signatures for these three liver cell populations (Table S6). The gene signatures of our four CAF subpopulations significantly intersected with those of these three liver cell types, suggesting phenotypic proximity between VSMCs and Ctr-CAF-I, between HSCs and Ctr-FAC-II, and between SAMes and ECM-CAFs (Table S7). We trained and evaluated different machine learning (ML) algorithms (random forest, support vector machine, and k-nearest neighbors) on the healthy/cirrhotic liver single-cell dataset 12. Since standard cross-validation indicated high accuracy (Table S8), we used these trained ML models with our CRC-LM CAF transcriptome data. This confirmed the phenotypic proximity between our populations and those of the liver atlas (Figures 2A and S14), which was also supported by RNA velocity analysis 22 (Figure 2B). Remarkably, a comparison of CAFs with their matched non-cancer mesenchymal cells showed a near-systematic increase in CAFs of the biological processes listed in Figure 1C (Figure S4-S9).

SAMes globally expressed PF markers, and a SAMes subpopulation was located in the periportal space 12. As our previous analysis showed ECM-CAFs proximity with the SAMes phenotype, we asked whether ECM-CAFs originated from PFs. First, we found that genes specifically express by PFs, but not HSC, in the liver were strongly associated with ECM-CAFs (Figures 2C and S15). Second, in CRC-LM adjacent to normal liver tissue, triple immunofluorescence (IF) staining of pan-cytokeratin (hepatocytes; Figure 2D, zone marked “a”), α-SMA (stellate cells/fibroblasts) and LTBP2 revealed a distinct enrichment of LTBP2+ cells in the portal regions of normal liver (Figure 2D, zone marked “b”). Third, LTBP2 staining coincided with typical aspects of collagen-containing connective tissue found in the portal space. We observed α-SMA staining particularly in stellate cells, in the Disse space. Conversely, we did not detect LTBP2 staining in the Disse space or hepatocytes.

We confirmed the expression of three signature genes specific to ECM-CAFs (*LTBP2*, *C3*, and *POSTN*) by RT-qPCR in relevant mesenchymal cells: a stellate cell line (LX2), fibroblasts (CCD18Co), and CAFs isolated from a CRC-LM sample (CRC-LM CAFs). First, we evaluated their expression levels in basal conditions (Figure 2E). *LTBP2* and *POSTN* were overexpressed in CRC-LM CAFs compared with LX2 and CCD18Co cells, while the opposite pattern was true for *C3*. This suggested that the CRC-LM CAFs (and following *in vitro* culture) were of the CP-CAF subtype. Furthermore, LX2 stellate cells tended to express less genes belonging to the ECM-CAF signature, which is compatible with a HSC origin of Ctr-CAF-II cells. To mimic the situation where mesenchymal cells are exposed to the cancer cell secretome, we incubated LX2, CCD18Co cells and CRC-LM CAFs with conditioned medium from three different CRC cell lines. This increased *C3* and *POSTN* expression only in CCD18Co fibroblasts (Figure 2F). The absence of upregulation in CRC-LM CAFs might be due to the very high basal levels of these two genes. *LTBP2* expression tended to be higher in CCD18Co fibroblasts exposed to conditioned medium, suggesting that the main regulation could be at the protein level. Western blotting of the conditioned media of the mesenchymal cells used for RT-qPCR confirmed this hypothesis (Figure 2G). We complemented the results in Figure 2E-G by comparing *LTBP2*, *C3* and *POSTN* abundance in the two main CAF populations and mesenchymal cells from 11 CRC-LM and 5 healthy liver samples (Figure S16). This analysis also showed higher expression in ECM-CAFs than in healthy HSCs and fibroblasts. This increase was less pronounced for Ctr-CAFs.

### ECM-CAFs accumulate at tumor locations with strong desmoplastic reaction

**Figure 3:**
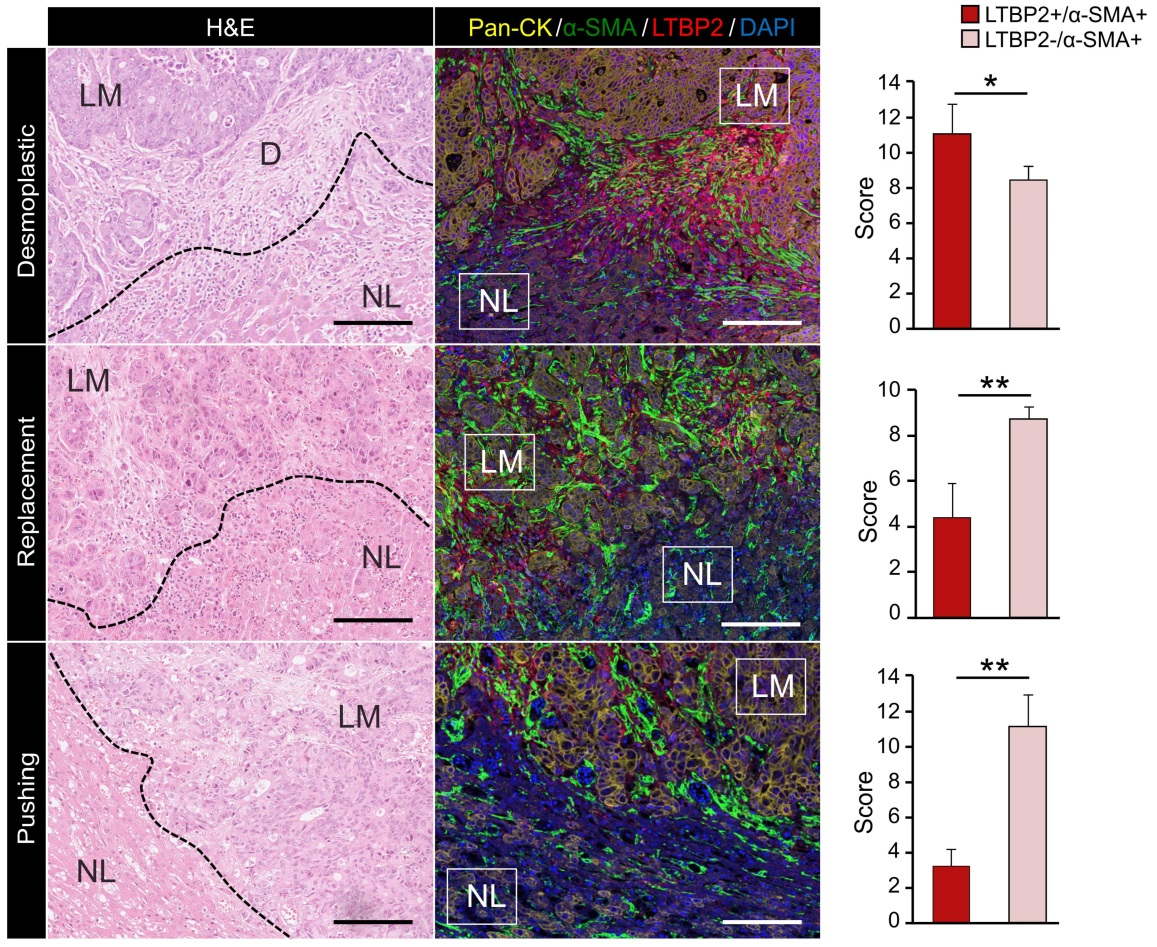
Expression of Pan-CK (cancer cells), α-SMA (CAFs) and LTBP2 (ECM-CAFs) in CRC-LM samples at the invasion front of replacement, and in the pushing and desmoplastic HGPs. LM, liver metastasis; D, desmoplasia; and NL, normal liver. Representative images of nine CRC-LM samples; scale bars, 100 μm. Nuclei were counterstained with DAPI. Histograms indicate the mean values from nine samples and two different locations *per* sample; error bars are the standard deviations; *P ≤ 0.05, **P ≤ 0.01 (Mann-Whitney-U-Test).

Bioinformatic analysis above indicated that ECM-CAFs were especially implicated in ECM remodeling. Thorough analysis of CRC-LM tissue samples stained with LTBP2 and α-SMA (Figure 1E) revealed that LTBP2+ ECM-CAFs tended to accumulate at areas of strong desmoplastic reaction. To confirm this initial observation, we explored the invasive fronts of nine CRC-LM samples (two locations *per* metastasis) that were classified based on their histologic growth patterns (HGPs) 23-25. In this classification, the transition zone where cancer cells grow towards normal liver parenchyma, the surrounding stromal cells, and the extracellular matrix (ECM) are taken into account to define three HGPs: i) desmoplastic (or encapsulated) HGP, characterized by extensive collagen deposition, prominent angiogenesis, and no contact between tumor cells and hepatocytes; ii) pushing (or expansive) HGP, devoid of desmoplastic reaction, tumor cells are separated from hepatocytes by a thin reticulin fiber layer, and liver cells are pushed away by the metastasis. A modest immune infiltrate can be present at the interface; iii) replacement HGP where cancer cells infiltrate the liver parenchyma without altering its structure, unlike the other two HGPs. All CRC-LMs can be classified using the HGP system; however, metastases frequently display more than one HGPs that are spatially separated. Analysis of IF images clearly showed that the proportion of ECM-CAFs was significantly higher at the invasive front of metastases with the desmoplastic HGP (Figure 3). We also considered CRC-LM samples with more than one HGP and each was analyzed separately. Independently of the invasive front and the HGP, the proportion of ECM-CAFs was higher in metastases with a strong desmoplastic reaction at their center (Figure 1E).

### ECM-CAFs and angiogenesis

**Figure 4:**
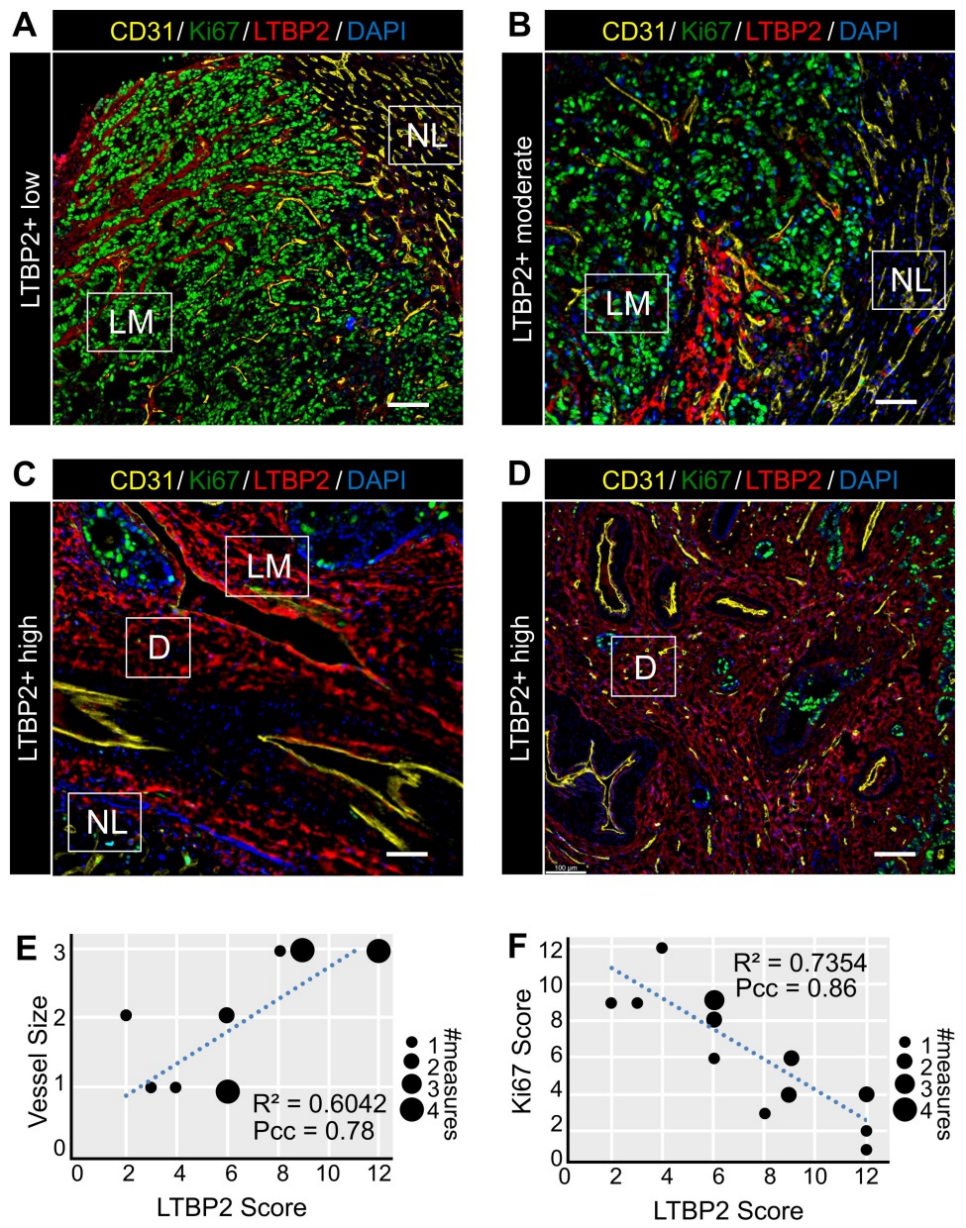
Features of ECM-CAF dense areas. **A-D.** Immunofluorescence analysis of endothelial cells (CD31), cancer cell proliferation (Ki67), and ECM-CAFs (LTBP2) in different configurations representative of 20 CRC-LM samples. **A.** ECM-CAF low concentration area at the invasive front of a CRC-LM with replacement HGP. **B.** Moderate ECM-CAF concentration at the invasive front of a CRC-LM with pushing HGP. **C.** High ECM-CAF concentration at the invasive front of a CRC-LM with desmoplastic HGP. **D.** High ECM-CAF concentration at the center of a CRC-LM with replacement HGP. LM, liver metastasis; D, desmoplasia; and NL, normal liver. Scale bar, 100 μm; nuclei were counterstained with DAPI. **E.** Correlation analysis of CD31+ vessel size (1-small, 2-medium, 3-large) and ECM-CAF (LTBP2+) presence. **F.** Correlation analysis of cancer cell Ki67-positivity and ECM-CAF (LTBP2+) presence. E and F: analyses restricted to LM areas, nine tumors, two different fields per tumor; Pcc = Pearson correlation coefficient, P = P-value.

As our bioinformatic analysis indicated an increased activity of angiogenesis-related pathways in ECM-CAFs (Figure 1C), we tested whether angiogenesis was correlated with ECM-CAF presence. To this end, we evaluated CD31 and LTBP2 expression in nine CRC-LMs (two locations each). Normal tissue/cancer interfaces in metastases with the replacement and pushing HGPs displayed ECM-CAF-poor areas, where IF analysis evidenced small, capillary-type vessels (Figure 4A-B), similar to those observed in normal hepatic parenchyma. Conversely, in highly desmoplastic areas, numerous large vessels (not capillaries) were readily observable (Figure 4C-D). This is consistent with previous reports that underscore the importance of *de novo* angiogenesis in the desmoplastic HGP 26. As observed for ECM remodeling, angiogenesis associated with ECM-CAF density at the center of metastases when it was concomitant with a strong desmoplastic reaction (Figure 4D). LTBP2 expression positively correlated with vessel size in all analyzed CRC-LM samples (Figure 4E).

### ECM-CAFs and cancer cell growth

Areas where ECM-CAFs were strongly present (as indicated by high LTBP2 expression) displayed a collagen-rich desmoplastic reaction (as evidenced by Saffron-Masson staining) (Figure S17). This observation was fully in line with the single-cell RNAseq data that clearly indicated significant upregulation of collagen-expressing genes in ECM-CAFs (Figure 1BC). Collagen leads to a more abundant and stiffer ECM, and this might hamper cancer cell growth. This hypothesis was supported by the negative correlation between Ki67 positivity in cancer cells and ECM-CAF abundance in CRC-LM tissues (Figure 4ADF).

### Mapping CAF-cancer cell interactions

**Figure 5:**
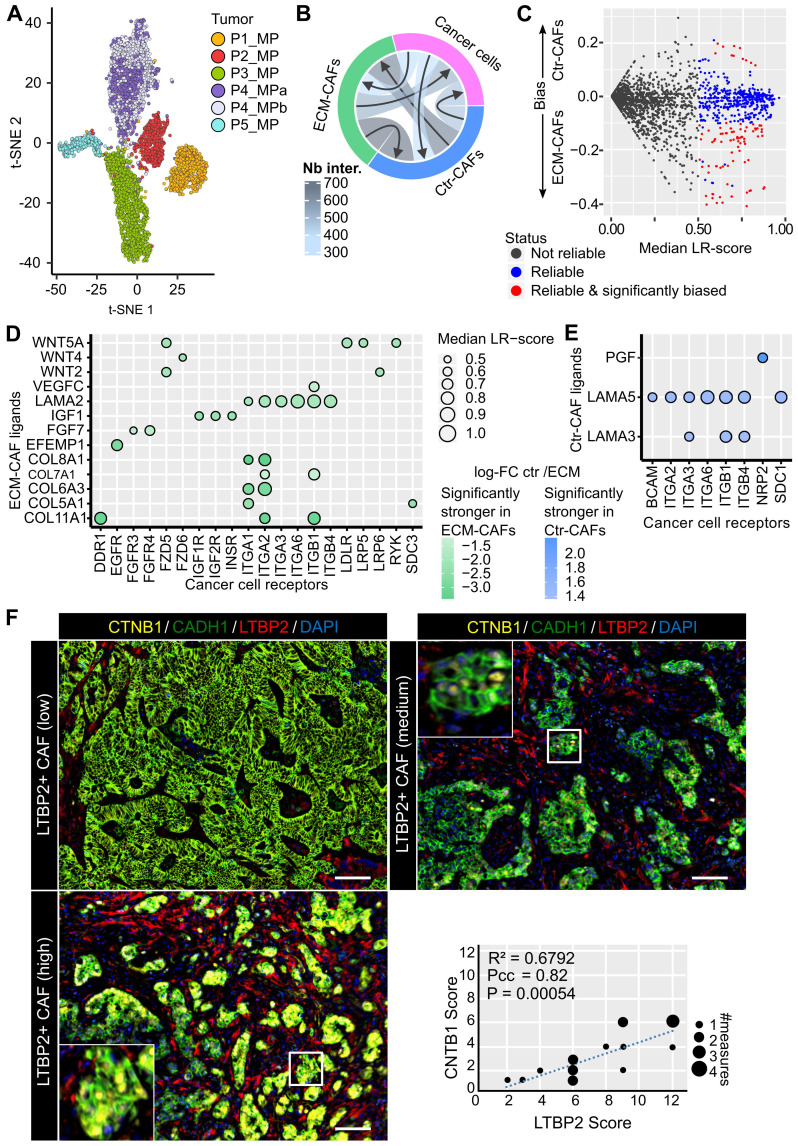
Cell interactions. **A.** Two-dimensional projection of the cancer cell transcriptomes. **B.** Number of reliable LR interactions among the three cell populations. **C.** Schematics of the selection of reliable and significantly Ctr- or ECM-CAF-biased LR interactions. Vertical axis: differences between the median LR-scores for Ctr- and ECM-CAFs. **D.** Chosen subset of significantly stronger LR interactions from ECM-CAFs to cancer cells. **E.** Significantly stronger LR interactions from Ctr-CAFs to cancer cells. **F.** Multiplexed immunofluorescence staining of cancer cells (CADH1), Wnt canonical signaling (CTNB1), and ECM-CAFs. Images show three liver metastasis zones with high, medium and low ECM-CAF proportions. Representative images of nine CRC-LM samples; scale bar, 100μm; nuclei were counterstained with DAPI. **G.** Correlation analysis of nuclear CTNB1 positivity in cancer cells and presence of adjacent ECM-CAFs: nine CRC-LMs and two different fields per sample; Pcc = Pearson correlation coefficient, P = P-value.

EPCAM+ cells from the six CRC-LMs (5,331 cells in total, see Table S1) formed well-defined clusters that correlated with the patient of origin (Figure 5A). Notably, the two metastases from patient 4 grouped together. EPCAM+ cell clusters uniformly expressed epithelial cancer cell markers and were devoid of cholangiocyte markers (Figure S18A-B). We confirmed the absence of *LTBP2* and *ACTA2* expression in EPCAM+ cells (Figure S18C).

To infer ligand-receptor (LR) interactions between cancer cells and the two main CAF populations, we employed SingleCellSignalR 27. In this tool, inferences are predicted by relying on a curated database of known *in vivo* and *in vitro* LR interactions and the computation of a score for each interaction, *i.e.*, LR-score. An LR-score above 0.5 indicates 95% confidence in the interaction 27. For LR interactions between cancer cells and CAFs, we computed six LR-scores, one *per* metastasis, and imposed the 0.5 threshold on the median (in general, LR-scores of different metastases were close to each other, data not shown). We found that the highest number of paracrine LR interactions occurred between CAFs, followed by CAF-to-cancer cell interactions, and cancer cell-to-CAF interactions (Figure 5B). Most of the molecules involved in these interactions were growth factors or molecules related to ECM, cell-cell interactions, or chemotaxis.

Next, we focused on the difference between interactions linking ECM- or Ctr-CAFs to cancer cells. For a given LR interaction, we computed the difference between its median ECM-CAF-to-cancer cell LR-score and its median Ctr-CAF-to-cancer cell LR-score. We set the following requirements: the difference between these median LR-scores needed to be > 0.1, and the genes encoding the featured ligands needed to be differentially expressed between ECM- and Ctr-CAFs (FDR < 1%, FC > 2) (Figure 5C). We found 78 significantly stronger interactions from ECM-CAFs to cancer cells (chosen subset in Figure 5D, full list in Figure S19), and 11 significantly stronger interactions from Ctr-CAFs to cancer cells (Figure 5E). Stronger interactions originating from ECM-CAFs included growth factors, Wnt signaling, and angiogenic interactions. In addition, Ctr- and ECM-CAFs seemed to modulate laminin trimers differently. The selection of significantly biased LR interactions relating cancer cells to the two CAF populations identified 37 stronger interactions from cancer cells to ECM-CAFs and 10 to Ctr- CAFs (Figures S20-S22). ECM-CAFs showed a larger number of stronger interactions with cancer cells in both directions.

Cell interaction inference suggested that ECM-CAFs were involved in Wnt/β-catenin signaling in cancer cells (Figure 5D). We sought to validate this observation at the protein level. We co-stained CRC-LMs with anti-LTBP2 and -β-catenin antibodies, and then evaluated the extent of nuclear β-catenin staining near ECM-CAFs. We could observe nuclear accumulation of β-catenin in such areas (Figure 5F). Correlation analysis confirmed the significant association between these proteins (Figure 5F).

### LTBP2 modulates the ECM-CAF phenotype

**Figure 6:**
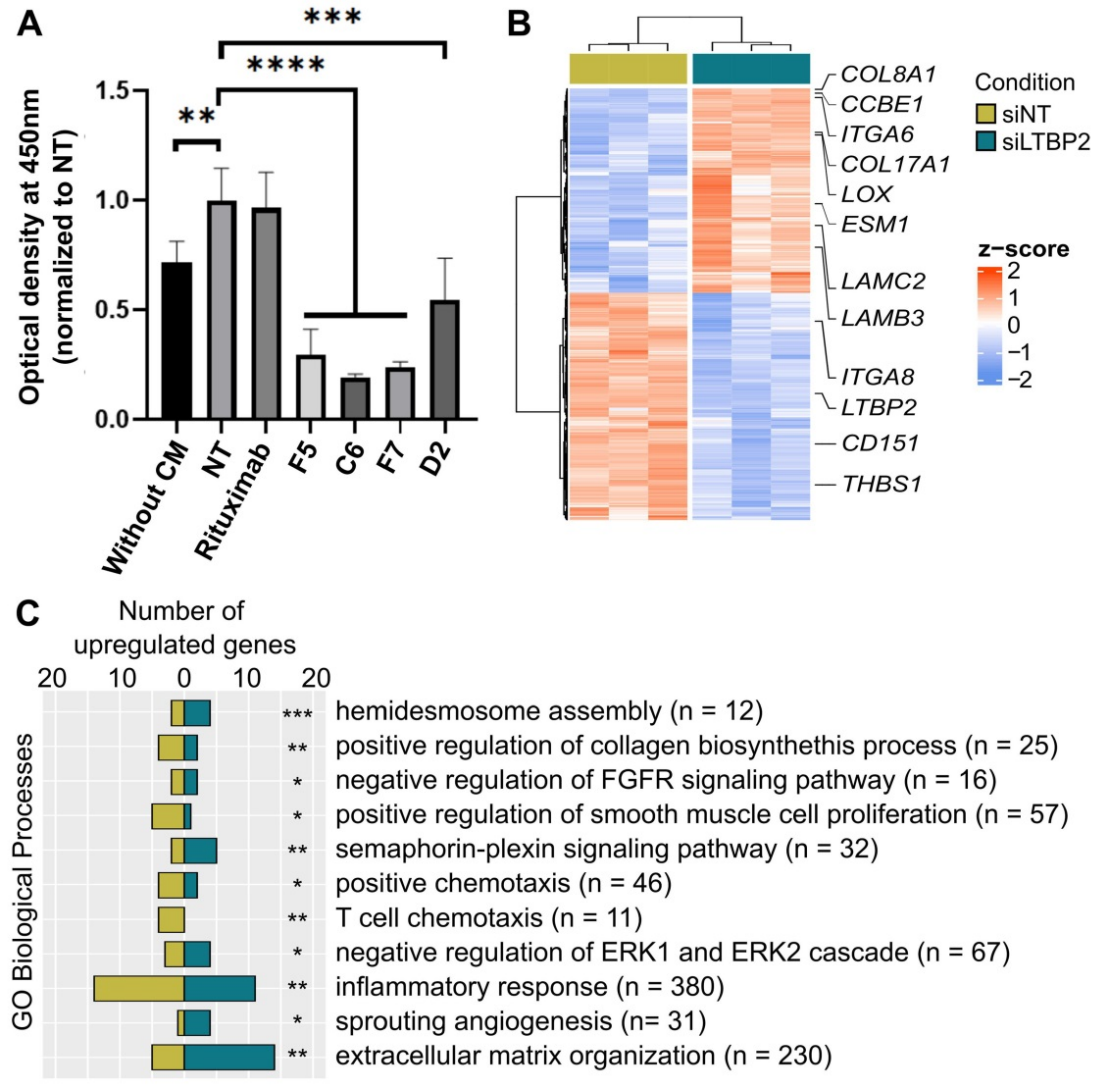
LTBP2 is essential for fibroblast viability and functions. **A.** MTT assay showing the proliferation of CCD18Co cells exposed to HT29 conditioned medium (CM) and incubated with different antibodies against LTBP2 (F5, C6, F7, D2), or an irrelevant antibody (rituximab, against CD20) for 96h. Mean values were normalized to the non-treated condition (NT). Error bars represent the standard deviation. P-values were obtained with the *t*-test. **B.** Expression (z-scores) of 496 genes significantly deregulated (edgeR analysis, FDR < 0.01 and fold-change > 2) between CCD18Co cells transfected with siRNAs anti-*LTBP2* and non-target (siNT) for 48h. **C.** Selected GO biological processes modulated by *LTBP2* silencing in CCD18Co fibroblasts. Panels A and C: *P < 0.05, **P < 0.01, ***P < 0.001, ****P < 0.0001 (hypergeometric test).

LTBP2 was among the strong markers of ECM-CAFs. It is a secreted protein that could be systemically reached and targeted *in vivo*. Therefore, we selected, by phage display, four fully human anti-LTBP2 antibodies (IgG) (Figure S23A-B). Incubation of CCD18Co fibroblasts with three of these anti-LTBP2 antibodies reduced significantly their viability (tested with the MTT assay) (Figure 6A). We did not observe any effect in HT29 CRC cells (data not shown). This was not surprising because LTBP2 expression in CRC is limited to fibroblasts/CAFs (Figures 1D and S11). To propose a possible mechanism of action in fibroblasts, we observed that already 96h after addition of the anti-LTBP2 antibodies, fibroblasts became round and started detaching from the well. Propidium iodide/Hoechst staining of the detached cells did not suggest apoptosis or necrosis (data not shown), thus implicating a subtler mechanism. Incubation of CRC-LM CAFs with the same antibodies showed that i) higher antibody amounts were needed due to the higher LTBP2 expression levels in CAFs; and ii) two antibodies induced CAF depletion (Figure S23C).

To obtain information on *LTBP2* role in fibroblast biology, we silenced *LTBP2* by siRNAs in which cells, and performed RNA-sequencing. We found 496 significantly deregulated genes, suggesting an important role (Figure 6B). Roughly half of the deregulated genes were upregulated upon *LTBP2* silencing, while the other half were downregulated. Biological process enrichment analysis identified several deregulated pathways. A selection of biological processes related to fibroblast biology and inflammation is reported in Figure 6C (full list in Table S9). Representative genes of some pathways are listed in Figure 6B: integrin- and collagen-encoding genes, *LOX* (involved in ECM collagen cross-linking and stiffness), and *CD151*, an essential gene for hemidesmosome formation and stability, and thus related to cell-ECM adhesion.

### Relation with liver primary tumors

**Figure 7:**
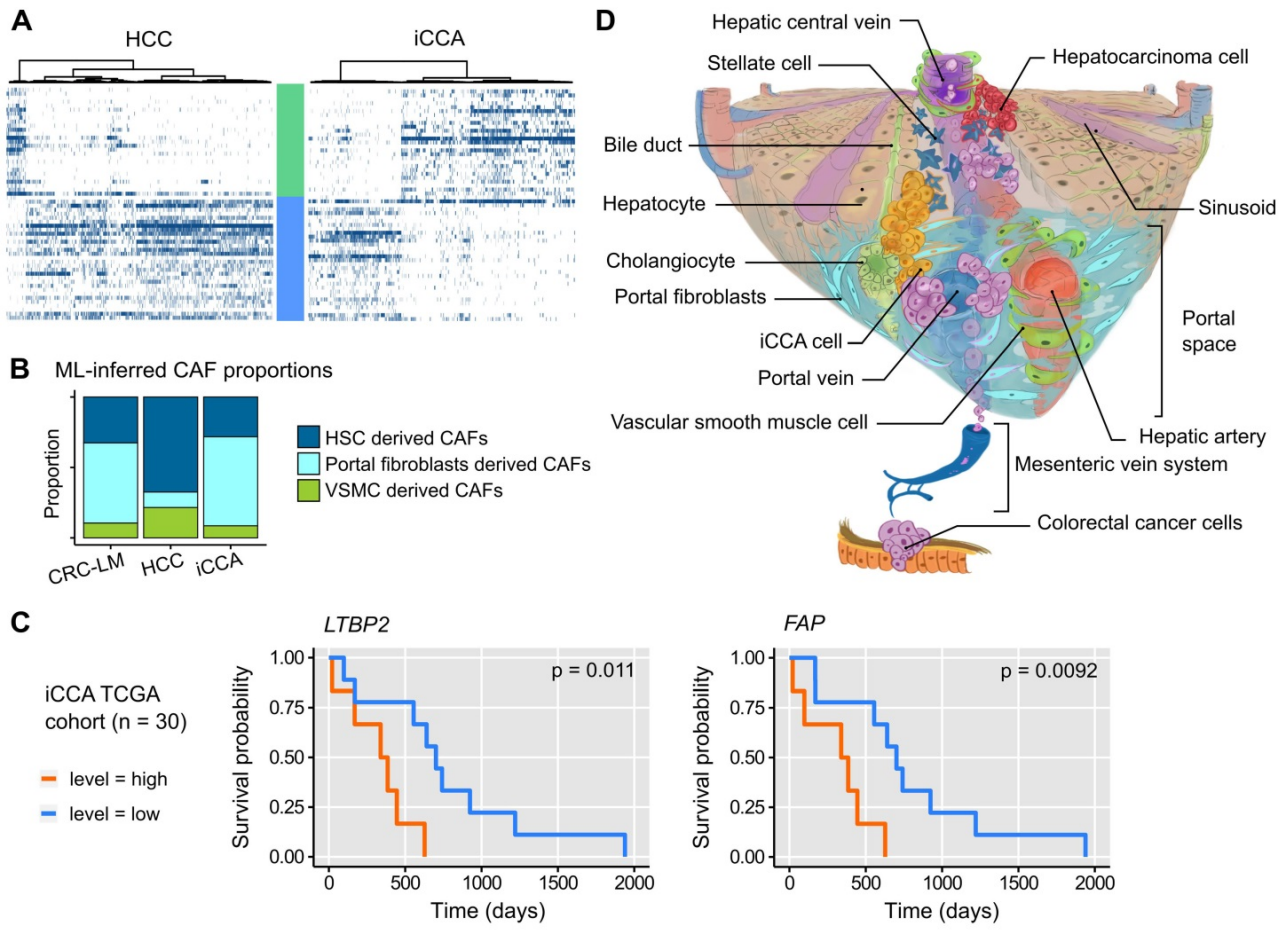
**A.** Expression profiles of ECM- and Ctr-CAF signature genes in different liver tumor types. **B.** ML-inferred CAF composition in CRC-LM, hepatocellular carcinoma (HCC), and intrahepatic cholangiocarcinoma (iCCA). P-values by multinomial testing (#P < 7E-11). **C.** Survival curves in function of *LTBP2* and *FAP* expression. **D.** CAF origin model. CAFs mainly derive from three major sources: hepatic stellate cells (HSC), portal fibroblasts (PF), and vascular smooth muscle cells (VSMC). CRC-LM initiate by the arrival of CRC cells from the colon through the portal vein into the portal space of liver. There, they extravasate, activate PFs, and orient them to a CAF phenotype. The lesions grow and invade the liver parenchyma where they subsequently convert HSCs into CAFs. In HCC, the opposite occurs. Tumors originate in the liver parenchyma where they first activate HSCs.

To determine whether CAFs similar to the ECM- and Ctr-CAFs in CRC-LM were present also in other liver lesions, we analyzed single-cell transcriptomes of hepatocellular carcinoma (HCC) and intrahepatic cholangiocarcinoma (iCCA) 28. The two major CAF populations (ECM- and Ctr-CAFs) were present in HCC and iCCA (Figures 7A and S24). The proportions of these two fibroblast populations were comparable in iCCA and CRC-LM; however, ECM-CAFs were less abundant in HCC. Using our ML model, we found that both iCCA and CRC-LM harbored comparable and significant proportions of PF-derived CAFs (Figure 7B). Conversely, HCC harbored mostly HSC-derived CAFs (Figure 7B). Using a cohort of 30 iACC specimens available in TCGA, we detected an association between overall survival and several ECM-CAF signature genes, notably *LTBP2* and *FAP* (Figure 7C; more genes in Figure S25).

## Discussion

CAFs are abundant and versatile stromal cells in many solid tumors. They derive from different cell populations and not only from resident fibroblasts. We analyzed six CRC-LMs from five different patients at single-cell resolution. Clustering analysis of 4,397 CAF transcriptomes revealed two main populations that we called ECM- and Ctr-CAFs. Both populations expressed typical CAF genes, such as genes encoding collagens and other ECM-related proteins, as well as various growth factors. The expression of genes involved in angiogenesis, ECM remodeling, and collagen-rich fibrosis was much stronger in ECM-CAFs. On the other hand, Ctr-CAFs displayed stronger contractility. These two major CAF populations could be further clustered in two subpopulations with more specialized phenotypes. Through a detailed comparison with single-cell reports that described CAF subpopulations in primary tumors 7, 14, liver tumors 16, and CRC-LM cell population changes upon chemotherapy 15, we identified overlapping CAF subpopulations. However, our study featured 20-fold more CAFs compared with the previous analysis of CRC-LMs 15, thus increasing our capacity to map their heterogeneity with high confidence.

Our data showed that CAF heterogeneity is mainly based on their distinct origins. We approached this question first with a ML approach that allowed us to relate CAFs in CRC-LM to the mesenchymal cells observed in healthy and cirrhotic human liver samples 12. Our ML-based analysis predicted that ECM-CAFs originate from PFs expressing many fibrotic collagens (SAMes in Ref. 12), and that the two Ctr-CAF subpopulations (Ctr-CAF-I and Ctr-CAF-II) derive from VSMCs and HSCs, respectively. These results are in agreement with previous studies that described CAF populations in CRC-LM 15, 16 and other tumors 7, 14, 29, 30 or tissues 12. Only, in one report 31, the authors proposed that HSCs are the main source of CAFs in CRC-LM. Our results and other literature data do not support this hypothesis. In two other (primary) liver tumor types (HCC and iCCA), we identified CAF populations related to the CRC-LM ECM- and Ctr-CAFs, possibly with a common origin. We found that both iCCA and CRC-LM harbor comparable and rather large proportions of PF-derived CAFs, while HCC stroma features HSC-derived CAFs predominantly. We suggest that these quantitative differences are explained by how these different tumors reach the liver parenchyma (Figure 7D). CRC metastatic cells enter the liver through the portal vein, whereas iCCA develops in bile-ducts that are situated in the portal space. Therefore, these two tumor types might recruit portal fibroblasts first, before reaching a sufficient mass to colonize the liver parenchyma and activate HSCs. On the other hand, HCC develops in the liver parenchyma, often in a context of HSC-derived fibrosis. HCC come into to contact with the portal spaces only at later stages.

Single-cell studies have allowed identifying a rather significant number of CAF subpopulations in different malignancies, but protein markers to discriminate these CAF populations are still missing. In the present work, we showed that LTBP2, which was already known as a stromal marker in CRC, is a strong marker of ECM-CAFs 32. *LTBP2* silencing in colon fibroblasts followed by RNA-sequencing revealed a large number of significantly deregulated genes (N=496), thus suggesting an important role in fibroblast biology, particularly in ECM adhesion and hemidesmosome formation. Hemidesmosomes are cell membrane structures that allow cell adhesion to a collagen/laminin-rich matrix. CAF adhesion to this matrix is important for the architectural remodeling of the TME and for cancer cell migration 33. Furthermore, CAFs need to adhere to a support in order to prevent anoikis. Here, LTBP2 targeting using specific antibodies dramatically reduced the fibroblast adherence and viability in 2D cell culture conditions. Although the underlying mechanisms needs to be precisely investigated, our data suggest that LTBP2 could be targeted to deplete ECM-CAFs with potential anti-tumoral effects, as demonstrated in a mouse model using another CAF-depletion method 31.

LR modeling underlined the potential activation of the Wnt/β-catenin pathway by ECM-CAFs in cancer cells. This was coherent with IF data showing that in cancer cells located in regions enriched in ECM-CAFs, canonical Wnt signaling was upregulated. As ECM-CAFs originate from resident liver fibroblasts, it is interesting to note that in hepatocytes, Wnt/β-catenin signaling can synergize with insulin signaling through IGF1R, LRP5, and LRP6 34, which were concomitantly enriched in ECM-CAF-cancer cell LR interactions. As Wnt/β-catenin is essential for metastasis and CRC progression, our data suggest that physiological interactions between hepatocytes and portal fibroblasts could be exploited by metastasizing CRC cells. Low Ki67 expression in these cancer cells indicates that Wnt/β-catenin signaling did not induce proliferation in these circumstances. This pathway can contribute to many other functions, including EMT or the renewal of cells which have reached a certain degree of stemness 35. In breast cancer lung metastases, stromal POSTN is crucial for cancer stem cell maintenance by increasing Wnt signaling 36, and LTBP2+/POSTN+ CP-CAFs constitute the majority of ECM-CAFs. In the genomic classification of CRCs, the CMS2 subtype is characterized by strong activation of Wnt target genes 37. CMS2 is the subtype with the best clinical outcome 37. Interestingly, we found that ECM-CAFs were abundant in tumor regions with extensive desmoplastic reaction. The CRC-LM-specific HGP classification 23-25, 38, 39 identifies a desmoplastic growth pattern that has the best prognosis 38 among HGPs.

In conclusion, this study complements recent analyses on CRC-LM CAFs at the single-cell resolution. The number of sequenced CAFs was more than one order of magnitude larger compared with the existing data and we provided a detailed analysis of CAF heterogeneity. The two main CAF subtypes (ECM- and Ctr-CAFs) featured specific functional differences, and we showed that ECM-CAFs originate from PFs. We found that LTBP2, a protein specific to ECM-CAFs, could be targeted by antibodies *in vitro*. This might create opportunities to remodel ECM-CAF-rich CRC-LMs. Although CAF depletion might not be sufficient to eradicate tumors 31, such remodeling might be combined with other therapies, *e.g.*, anti-angiogenic compounds, as already explored in CRC 40. Moreover, in iCCA, ECM-CAF proportion was similar to that of CRC-LMs, and several genes expressed by ECM-CAFs, such as *LTBP2,* were associated with survival.

## Materials and Methods

### Patient Material

The Translational Study Committee of the Regional Cancer Hospital ICM, Montpellier approved this study. In accordance with the French law, patients who did not oppose to the use of their material for research purposes have provided consent (opting-out rule). Liver metastases from 14 patients with colorectal cancer (CRC-LM) were used in the present study. All patients were treated with neo-adjuvant chemotherapy before surgery and sample collection. Six different tumors were analyzed from five patients using single-cell RNA sequencing. Four patients had mono-focal liver metastases, while one had bi-focal metastases. The other CRC-LM samples were used in the validation study.

### Tumor sample collection and dissociation

Fresh primary tumor and liver metastases were cut into multiple pieces (10-15 mm^2^), while avoiding the surrounding non-tumoral tissue and visible necrotic areas. Then, tumor samples were reduced in size using surgical scissors and washed with cold Hanks' Balanced Salt Solution (cat. no. 14025092, Gibco, Thermo Fisher Scientific, Waltham, MA, USA). To 200 mg of sample, 8 mL of enzyme digestion mix was added. This mix included 1 mL of collagenase (20 mg/ml, cat. no. 0130, Sigma Aldrich, St. Louis, MI, USA), 1 mL of hyaluronidase (20 mg/ml, cat. no. H3506, Sigma Aldrich), 2.5 µL of DNase (100 µg/µl, cat. no. D5025, Sigma Aldrich) in 8 mL of RPMI medium (cat. no. 21875042, Gibco). For further details, see Supplemental Material.

### Cell sorting

Three to ten million cells were transferred to a clean 15 mL conical tube, and the volume was adjusted to 1 mL using 0.5% BSA/PBS solution. Next, five tubes each containing 100,000 cells in 100 µL 0.5% BSA/PBS solution were prepared for individual staining/negative control. The following antibodies/dyes were used (according to the manufacturers' instructions): anti-EPCAM-PE (cat. no. 347198, Becton Dickinson (BD), Franklin Lakes, NJ, USA), anti-CD31-Alexa488 (cat. no. 558068, BD), anti-CD45-APC (cat. no. 560973, BD) antibodies, and Live-Dead-NearIR dye (cat. no. L34961, Life Technologies, Thermo Fisher). The antibody-sample mix was incubated at room temperature (RT) for 30 min, then the 0.5% BSA/PBS solution was added to reach 10 mL, and samples were centrifuged at 300 xg at 4 °C for 5 min. Cells were suspended in 1 mL of 0.5% BSA/PBS solution and sorted using a FACS Aria 2 (BD). For further details, see Supplemental Material.

### Single-Cell RNA sequencing

Samples were processed following the 10x Genomics Single Cell 3' Reagent Kit v3 (10X Genomics, Pleasanton, CA, USA) user guide. Briefly, starting with the cell suspension, Gel Bead-In Emulsions (GEM) were generated, barcoded, and the reverse transcription reaction was performed. Purified cDNA was amplified for 12 cycles, and the resulting cDNAs were run on a Fragment Analyzer (High Sensitivity kit NGS) (Agilent Technologies, Santa Clara, CA, USA) to determine their quantity. cDNA libraries were prepared, adjusting the PCR cycles based on the calculated cDNA concentration, and using Chromium Single Cell 3' Library and Gel Bead Kit v3, Chromium Single Cell 3' Chip kit v3, and Chromium i7 Multiplex. The proportion of each library was calculated based on a preliminary shallow sequencing run using MiniSeq (Illumina, San Diego, CA, USA) and Mid Output Reagent Cartridge (Illumina). After evaluating the number of cells, reads and sequencing saturation, libraries were pooled in two-three samples per run and normalized to a final loading concentration. Each sequencing run was performed on NovaSeq using v1 chemistry. A sequencing depth of 50,000 reads/cell was targeted for each sample. Sequencing fastq files that passed the Illumina quality control criteria were analyzed using the 10X Genomics CellRanger pipeline, v 3.0.2 and 3.1.0.

### Data preparation and initial filtering

Raw data were processed using the 10x Genomics Cell Ranger software (v3.0.2). For each sample, the cells with top 0.05% or total UMIs were considered as doublets and were removed. Cells with <1,000 distinct genes measured were also discarded. Unless specified otherwise, each single-cell transcriptome was normalized to the total UMI count (division by the total and multiplication by 10^4^), and log-transformed (log_2_(1 + norm UMI count)). For further details, refer to Supplemental Material.

### Two-dimensional projections and clustering

EPCAM+ and TN cell 2-dimensional projections were obtained separately, using the 1,500 most variable genes (coefficient of variation) among the 5,000 most expressed genes in the respective cell populations. Only genes expressed in at least 1% of cells were considered. Then, the first 30 principal components identified by principal component analysis (PCA) were submitted to t-SNE (perplexity = 30). TN cells were clustered by computing the Euclidean distances between transcriptomes and constructing a dendrogram using the Ward's method. This computation used the same 1,500 genes as projection.

### Differential gene expression analysis and gene signatures

Differentially expressed genes were identified using edgeR 41. For this purpose, TMM normalization was applied by the calcNormFactors function, and the glmFit and glmLRT functions were used with default parameters to identify differentially expressed genes. P-values were corrected with the Benjamini-Hochberg procedure. Signature genes for each of the four TN cell clusters (CS-CAFs, CP-CAFs, Ctr-CAF-I, Ctr-CAF-II) were selected if they were expressed in at least 20% of the cluster cells, had a fold-change (FC) ≥2 and an adjusted P-value ≤ 1% compared with the other three clusters pooled together, and in the three comparisons against each cluster. The top 30 genes were kept, sorted according to their FC. The signature genes for the two main TN clusters (Ctr- and ECM-CAFs) were identified by requiring expression in at least 20% of the main cluster cells, and in at least 10% of each of the subcluster cells (*e.g.*, in Ctr-CAF-I and Ctr-CAF-II), a FC ≥ 2 and an adjusted P ≤1% compared with the other main cluster (Ctr- *versus* ECM-CAFs) and with its subclusters (*e.g.,* Ctr-CAFs *versus* CS- or CP-CAFs). The top 30 genes were kept and sorted according to their FC.

### RNA velocity

Loom files were generated for the TN fraction of each tumor with velocyto 0.17.17 and filtered and combined with loompy 2.0.16. Then, RNA velocity was analyzed with scvelo 0.2.4. For this, the function pp.filter_and_normalize was first applied to normalize and log-transform the data. Only 2000 genes were selected for the subsequent analysis (min_shared_counts = 20; n_top_genes = 2000). Spliced and unspliced counts two first order moments were computed with the function pp.moments (default parameters). Then, the dynamical model was run with the functions tl.recover_dynamics, tl.velocity (mode = “dynamical”) and tl.velocity_graph. Finally, velocities were projected on the tSNE with pl.velocity_embedding_stream.

### Non-cancer mesenchymal liver cells

Mesenchymal single-cell transcriptomes in liver were retrieved from a published atlas based on four healthy and three cirrhotic human livers 12. Among these cells, the authors identified four clusters, Mes(1), Mes(2), Mes(3), and Mes(4). Mes(4) was discarded in our study because it was identified as mesothelial cells. Then, the Mes(1-3) gene signatures were constructed following the same procedure as above: expression in at least 20% of cells of a given cluster, FC ≥ 2, and adjusted P ≤ 1% compared with each other cluster, and with the other two clusters together. As fewer genes met these criteria in this dataset, the signature sizes were based on the top 16 genes according to their FC.

### Machine learning approach

Three different models were built to classify new cells in one of the clusters described by Ramachandran, *et al.*: Mes(1), Mes(2), or Mes(3) mesenchymal cell subtypes 12. Random forest (R randomForest package, default parameters), K nearest neighbors (R DMwR package, function kNN, k = 100, norm = FALSE), and support vector machine (R package caret; trainControl with method = ”repeatedcv”, number = 10, repeats = 3; train with method = ”svmLinear”, preprocess = c(“center”,”scale”), tuneLength = 10) were used. The algorithm performance was evaluated by cross-validation using 90% of the cells to train the models and 10% to test them, repeated 20 times.

### Histology analysis

Formalin-fixed paraffin-embedded CRC-LM tissue sections were deparaffinized in xylene, and rehydrated in a series of methanol dilutions (95% to 50%). For staining with the hematoxylin-eosin Saffron dye, slides were immersed first in Mayer's hematoxylin (MHS16, Sigma Aldrich) for 30 sec, rinsed in tap water, and immersed in the eosin solution (HT110116, Sigma Aldrich) for 30 sec, followed by several alcohol baths to remove excess stain. Finally, slides were immersed in pre-warmed (40°C) Saffron solution (10047028, VWR) for 10 min. For the Masson's trichrome stain, following wax removal and rehydration, slides were stained using the Trichrome-Stain Kit (HT15-1KT, Sigma Aldrich) according to the manufacturer's recommendations. Once stained, all slides were rinsed in several baths of xylene, air dried, mounted with DPX (06522, Sigma Aldrich), and covered with a glass coverslip.

### Multiplexed immunofluorescence analyses

Formalin-fixed paraffin-embedded CRC-LM tissue sections were deparaffinized in xylene, and rehydrated in a series of methanol dilutions (see above). Antigen retrieval was done with the AR6 buffer (Perkin Elmer, Waltham, MA, USA; cat. no.: AR600250), and then sections were incubated in serum-free blocking solution for 30 min (Agilent-Dako, Santa Clara, CA, USA; cat. no.: X0909). Sections were then incubated with the primary antibody at 4 °C overnight, washed in PBS, and incubated at RT with the secondary antibody Histofine MAX PO Multi (Nichirei, Tokyo, Japan; cat. no. 414152F) for mouse and rabbit antibodies and Histofine MAX PO G (Nichirei Bio, cat. no. 414162F) for antibodies of goat origin, for 30 min. The following primary antibodies were used: anti-Ki67 (cat. no.: M724029-2, Dako), anti-CD31 (cat. no.: IR61061-2, Dako), anti-Pan-CK (cat. no.: GA05361-2, Dako), anti-β-catenin (cat. no.: M353901-2, Dako), a-smooth muscle actin (cat. no.: GA61161-2, Dako), anti-CADH1 (cat. no.: GA05961-2, Dako), and LTBP2 (cat. no.: AF3850, R&D Systems, Minneapolis, MN, USA). Subsequently, sections were washed in PBS (3 times for 5 min), and stained using the Opal system (Perkin Elmer, cat. no.: NEL810001KT). For further details, see the Supplemental Material section.

### Cell culture

The HT29, LOVO and CCD18Co cell lines were from ATCC (Virginia, USA). LX2 cells were from the Japanese Collection of Research Bioresources Cell Bank. SW1222 cells were a kind gift by Prof. W. Bodmer, Department of Medical Oncology, Weatherall Institute, Oxford, UK. The CRC-LM CAF cell line was isolated from a CRC-LM specimen. All cell lines were grown in Dulbecco's Modified Eagle Medium (DMEM) supplemented with 10% fetal bovine serum (FBS) and 1% penicillin/streptomycin (all from Gibco, Thermo Fisher Sci., Waltham, MA, USA) at 37 °C in 5% CO_2_.

Conditioned medium (CM) from CRC cell lines was obtained after 48h incubation of 80% confluent cells in serum-free DMEM. CM were collected, centrifuged at 150 g at RT for 5 min, and then added to CCD18Co, LX2 and CAF cell monolayers (cells were pre-starved in serum-free medium for 6h) for 48h. The control consists in the addition of serum-free DMEM. Then, CM medium was collected for Western blot analysis and processed in the same way as previously. Cell monolayers were washed with PBS twice and lysed for RNA extraction.

Human anti-*LTBP2* siRNA (ON-TARGET plus Human LTBP2 (4053), catalog no. L-011078-00-0005) and control siRNA (ON-TARGET plus Non-targeting Pool, catalog no. D-001810-10-05) were from Dharmacon (Lafayette, USA). CCD18Co cells were transfected with 40nM of siRNA using Lipofectamine (Lipofectamine 2000 reagent, catalog no. 11668-019, Life Technologies, Carlsbad, CA, USA). After 48h, cell monolayers were washed with PBS twice and lysed for RNA extraction.

### Phage-display selection of LTBP2 antibodies

Four different anti-LTPB2 antibodies were selected by phage display on recombinant human His-tagged LTBP2. His-tagged LTBP2 were produced by cloning the LTBP2 ORF (Cat. No.: OHu107637, GeneScrip, Piscataway, NJ, USA) in the pCMV vector (Cat. No.: 212220, Agilent, Santa Clara, CA, USA) and by transient transfection in HEK-293 T cells. Following HEK-293 T culture in standard conditions, medium was collected, centrifuged, and recombinant LTBP2 was purified by Ni-chromatography (Cat. No.: A50585, Thermo Fisher Sci., Waltham, MA, USA). Genes encoding the variable regions of the antibodies were provided into a modified pEF vector (Invivogen) allowing the expression and secretion of a fully human IgG1 in mammalian cells. Further details on antibody purification and characterization are provided in the Supplemental Material section.

### Cell incubation with antibodies and MTT assay

CCDC18Co cells and CAFs were grown in DMEM in standard conditions, and CM from HT29 cancer cells were obtained as described above. For the experiments, CCD18Co cells and CAFs were seeded on day 1 at 4000 cells/well in 96-well cell culture plates (catalog no. 83.3924.005, Sarstedt, Nümbrecht, Germany) with DMEM and 10% FBS. Twenty-four hours later, medium was removed and cells were washed once with PBS. Then, 150 μL of CM/DMEM mix was added to CCD18Co cells. The mix contained 50% of fresh DMEM and 50% of CM from HT29 cells starved for 48h. To this mix, 1% FBS (final concentration) was added. Next, antibodies were added (10μg/mL for CCD18Co cells and 100μg/mL for CAFs). Then, cells were incubated at 37ºC and 5% CO_2_ for 120h. Afterwards, cell viability was assessed using 3-(4, 5-dimethyl thiazol-2-yl) 2, 5-diphenyl tetrazolium bromide) (MTT) staining (catalog no. M5655, Sigma-Aldrich). Absorbance was measured at 540 nm. Statistical analysis was performed using the *t*-test.

### Western blot analysis

CM were concentrated 10-fold using Vivaspin columns 10 kDa filters (catalog no. VS0102, Sartorius Stedim Biotech, Stonehouse, UK). The cell culture medium was exchanged with RIPA buffer (150 mM NaCl, 0.5% Na-deoxycholate, 1% Triton X-100, 0.5% SDS, 50 mM Tris-HCl (pH 7.5)). Laemmli buffer (0.1% 2-mercaptoethanol, 0.0005% bromophenol blue, 10% glycerol, 2% SDS in 63 mM Tris-HCl (pH 6.8)) was added to 20 µl of concentrated CM. CM were then boiled for 5 min and loaded (identically amounts) on two 6% polyacrylamide gels. One of the gels were used for western blotting after protein transfer to nitrocellulose membranes at 100 V for 2 h. After blocking in 5% skim milk for 1h, membranes were incubated (4 °C, overnight) with an anti-LTBP2 antibody (1:500; catalog no. AF3850, R&D systems, Minneapolis, USA). The second gel was used for normalization after staining using the PlusOne Silver Staining Kit, Protein (catalog no. 17-1150-01, GE Healthcare, Uppsala, USA).

### Gene expression analysis by real-time reverse transcription quantitative PCR (RT-qPCR)

Total RNA was isolated with the Monarch Total RNA Miniprep Kit (catalo no. T2010S, New England Biolabs). For RT-qPCR analysis, RNA was reverse-transcribed using SuperScript III Reverse Transcriptase (catalog no. 18080; Invitrogen, Carlsbad, CA, USA). Twenty nanograms of cDNA was used for PCR reactions.

The basal gene expression levels were assessed using three biological replicates for CAFs and LX2 cells and four replicates for CCD18Co fibroblasts. Ct values were compared using the unpaired Welch's test and the t.test R function (var.equal = FALSE).

CM effect was evaluated in two (CAFs), three (LX2 cells) and four (CCD18Co cells) biological replicates. For each cell line, ∆Ct (relative to 18S) values were compared between conditions with the paired Student's *t*-test (var.equal = FALSE). Fold changes compared with control condition are reported.

### Bulk RNA sequencing

Total RNA was isolated as previously described. For each sample, 1µg of total RNA was used to construct the sequencing libraries. Libraries were prepared with the RNA Stranded Total RNA prep Ligation with Ribo-Zero plus kit (Illumina, San Diego, USA) to deplete ribosomal RNA. Then, they were sequenced on a NovaSeq6000 SP (200 cycles) (Illumina) to generate 66 million reads in each direction per sample. Base calling and demultiplexing steps were performed using the Illumina software Dragen 3.8.4. With our pipeline, Fastq files were aligned against the human genome (Ensembl GRCh38) (STAR using default parameters and 2 passes, read counts extraction with HTSeq-count).

TMM normalization was applied with the calcNormFactors function (edgeR R package), and the glmFit and glmLRT functions were used with default parameters to identify differentially expressed genes. P-values were corrected with the Benjamini-Hochberg procedure (multtest R package). Normalized transcriptomes were then log-transformed (x → log2(x+1)), and z-scores were computed.

The functional analysis of the differentially expressed genes (adjusted p-value <0.01, fold change absolute value >2) was performed with hypergeometric tests on Gene Ontology biological processes (GOBP) containing at least three differentially expressed genes.

### Data access and sample IDs

Single-cell transcriptomes are available from GEO (reference GSE158692). In these data, patient 1 metastasis (P1_MP) is referenced as SC_196081, P2_MP as 19G00619, P3_MP as 19G00635, P4_MPa as 19G02977_Big, and P4_MPb as 19G02977_Small, and P5_MP as 20G00953. RNA-sequencing data from cultured cells are available from GEO (reference GSE191323).

## Supplementary Material

Supplementary figures and tables.
