# Supplementary material for: The landscape of cancer-associated fibroblasts in colorectal cancer liver metastases
Source: Theranostics. 2022 Oct 31;12(17):7624–39. doi: 10.7150/thno.72853 (PMC9691344; doi:10.7150/thno.72853)
Supplement: Supplementary file 1 — Supplementary figures and tables. [file thnov12p7624s1.pdf]

Supplementary Material

The cancer-associated fibroblasts landscape in colorectal cancer-liver metastases

Ambre Giguelay[1,2,3,4], Evgenia Turtoi[1,2,4], Lakhdar Khelaf[5], Guillaume Tosato[1,2,3], Ikrame Dadi[1,2,4], Tommy Chastel[1,2,4], Marie-Alix Poul[1,2,4], Marine Pratlong[6], Stefan Nicolescu[1,2,4], Dany Severac[6], Antoine Adenis[7], Olivia Sgarbura-Popescu[8], Sébastien Carrère[8], Philippe Rouanet[8], François Quenet[8], Marc Ychou[7], Didier Pourqier[1,2,4,5], Pierre-Emmanuel Colombo[6], Andrei Turtoi[1,2,4,9]†, Jacques Colinge[1,2,3,]†

[1]Institut régional du Cancer de Montpellier (ICM)-Val d'Aurelle, Montpellier, France.
[2]Université de Montpellier, Montpellier, France.
[3]Cancer Bioinformatics and Systems Biology Team, INSERM U1194, Montpellier, France.
[4]Tumor Microenvironment and Resistance to Treatment Lab, INSERM U1194, Montpellier, France.
[5]Department of Pathology, Institut régional du Cancer de Montpellier (ICM)-Val d'Aurelle, Montpellier, France
[6]Biocampus, CNRS, INSERM, Université de Montpellier, Montpellier, France.
[7]Department of Medical Oncology, Institut régional du Cancer de Montpellier (ICM)-Val d'Aurelle, Montpellier, France
[8]Department of Surgery, Institut régional du Cancer de Montpellier (ICM)-Val d'Aurelle, Montpellier, France
[9]Gunma University Initiative for Advanced Research (GIAR), Maebashi, Gunma, Japan

Running title: LTBP2+ CAF in Liver Metastases.

† Equal contribution/co-corresponding authors.
Jacques Colinge, PhD
Email: jacques.colinge@inserm.fr
Tel : +33.(0)4.11.28.31.18
Andrei Turtoi, PhD
Email: andrei.turtoi@inserm.fr
Tel: +33.(0)4.11.28.31.88

Supplementary Methods

Tumor Collection and Dissociation

Tissue fragments with the enzyme mix were placed on an agitator at 37 °C and incubated for 45 min. Following this, the mixture was filtrated using 70 µM and then 30 µM mash size strainers (cat. no. 130-098-462 and 130-098-458, Miltenyi Biotec, Bergisch Gladbach, Germany). The undigested tissue pieces were returned to the flask, while the cells were separated from the digestion medium by centrifugation (5 min at 300 x g, 4 °C). The cell pellets were immediately washed twice in cold PBS (cat. no. 10010023, Gibco) containing 0.5% BSA (cat. no. A3294, Sigma Aldrich). Red blood cells were lysed by incubation 1 min at room temperature in 1ml of Ammonium-Chloride-Potassium (ACK) lysis buffer (cat. No A1049201, Gibco). The reaction was immediately stopped with 10ml of cold PBS 0.5% BSA. The cell pellets were then washed in cold PBS 0.5% BSA. In parallel, the cell-free digestion medium was returned to the undigested tissues for a second round of digestion (45 min and 37 °C). Following this second digestion, cell viability was estimated in both cell populations using trypan blue staining (cat. no. 15250061, Gibco). Minimal acceptable viability for subsequent experiments was 30%. Samples from 1st and 2nd digestions were pooled if of similar viability; otherwise samples from the 2nd digestion were used, as their viability was generally higher.

Cell Sorting

Compensations and auto-fluorescence was checked using individual stains. The following populations were isolated: cancer cells (EPCAM+/CD45-/CD31-/LiveDead-) and cancer associated fibroblasts (CAF) (EPCAM-/CD45-/CD31-/LiveDead-). After counting, 10.000 cancer cells and 10.000 CAFs were separately resuspended in 0.05% BSA PBS solution, to produce cell suspensions at approximately 1300 cells/µL. The cells suspensions were used immediately after the sorting for single-cell RNAseq library preparation.

Data preparation and initial filtering

Patient 5 (sample ID 20G00953M) EPCAM+ cells were pooled with CD45+ cells before sequencing. We did not use the CD45+ cells in this study. EPCAM+ cells were identified by performing PCA on all the genes detected in at least 1% of the cells, keeping the first 30 principal components, and using K-means clustering (k=3). CD45+ cells grouped in one unique cluster and were discarded.

Two-dimensional projection of all the EPCAM+ fractions (all the 6 tumors) together revealed a small cluster of cells separated from the main clusters grouping EPCAM+ cells of each patient (data not shown). In the liver, cholangiocytes also express EPCAM along with KRT7, KRT19, and SOX9.[1] Most of the cells in this cluster expressed KRT7, which was not expressed in the main EPCAM cell clusters. The other genes were not specific. To circumvent dropouts in KRT7 expression, we constructed a gene signature comprised of the 10 most KRT7-correlated genes in the cholangiocyte

cluster. Average expression of those 10 genes defined a cholangiocyte score, and EPCAM+ cells scoring ≥ 1 were discarded (61 cells, see Suppl. Fig. 1A).

Due to the absence of a generic surface marker, CAF were searched using a triple negative (TN) selection in flow-cytometry. In the TN fraction, we eliminated contaminating hepatocytes by using ALB as a marker. To address dropouts as above, we computed the 10 most ALB-correlated genes (84 cells removed, Suppl. Fig. 1B). Similarly, contaminating immune cells were identified with CD45 (17 cells removed, Suppl. Figure 1C). Contaminating epithelial cells were eliminated using the same procedure, and EPCAM as a marker (48 cells removed, Suppl. Figure 1D). Lastly, endothelial cells were eliminated from the TN fraction using the expression of CD31 (PECAM1) alone, since no well-correlated genes were found (66 cells removed, Suppl. Fig. 1E). Because the metabolic activity can be highly dysregulated in a tumor environment, a maximum content of 50% mitochondrial genes was allowed for TN cells.

Heatmaps

Heatmaps were generated with ComplexHeatmap.[2] Thresholds to the top and bottom 2.5% values were applied to obtain informative color scales.

Multiplexed immunofluorescence

The Opal staining solution was made by mixing 2 μL Opal dye and 98 μL Amplifying Buffer (100 μL total per slide). Following a 10 min incubation, the slides were washed three times for 5 min in PBS and then subjected to microwave-assisted antibody removal. Slides were immersed in AR6 buffer and the antibodies were striped using microwave for 15 min. Following this, the tissues were re-blocked with serum-free blocking solution for 30 min at RT. Tissues were then incubated with the next primary antibody, and the staining procedure repeated as described above using the following Opal dyes: 520, 570, 620 and 690. Stained slides were mounted using VECTASHIELD® Antifade Mounting Medium with DAPI (Vector, Burlingame, USA) and visualized on a Vectra Polaris fluorescence slide scanner (Akoya).

Scoring of the immunofluorescence was conducted using a semi-quantitative approach by two independent evaluators of two areas per section and patient. For a given target, two parameters were evaluated and later multiplied to yield a composite value (here referred to as score). The first parameter was the extent of staining with 1 denoting less than 25% of the surface occupied by target cells, 2 between 25 and 50%, 3 between 50 and 75% and 4 for 100%. The second parameter was the intensity of the staining in the target cells, with 0 denoting negative staining, 1 low, 2 medium and 3 high staining intensity.

Anti-LTPB2 antibodies

Human IgG1 obtained after plasmid transient transfection of HEK-293 T cells were purified using protein A affinity chromatography, as previously described (Le Gall M, Crepin R, Neiveyans M, Auclair C, Fan Y, Zhou Y, et al. Neutralization of KIT Oncogenic Signaling in Leukemia with Antibodies Targeting KIT Membrane Proximal Domain 5. Molecular cancer therapeutics 2015; 14:2595-605). Purified IgG1 were concentrated and buffered in PBS using Vivaspin 500 (MWCO 10 kDa) centrifugal Filter Units (Sartorius), quantified by spectrophotometry (1 uOD280nm = 0.75 g/L), filtered for sterility (0.22 µm Minisart Syringe filters, Sartorius) and purity was checked by a 10% SDS-PAGE (5 µg) and stained with Coomassie-Brilliant Blue G-250. Antibodies preparations were conserved in PBS at 4°C after sterile filtration.

Indirect Enzyme-linked immunosorbent assay (ELISA)

Maxisorp 96-well plates (Nunc) were coated with recombinant LTBP2 (100 ng/well in PBS) overnight at 4°C. The residual-binding sites were blocked with 2% (w/v) skim milk powder in PBS (MPBS, 200 µl/well). The antibodies were diluted in MPBS and titrated 1 to 10 in duplicates starting from 100 nM and incubated for 2 h at RT. Bound antibodies were detected by incubation with HRP-conjugated antibodies specific for human Fc (Sigma) (one hour at RT) followed by incubation with enzymatic chromogenic substrate solution Step-ULTRA-TMB (Thermo Scientific). Enzymatic reaction was terminated using 100 µl of 1 M HCl and OD at 450 nm was measured. In general, the plates were washed three times with PBST (PBS + 0.005% (v/v) Tween 20) and once with PBS in between each incubation step. Rituximab, an anti-CD20 of the IgG1 isotype (Roche) was used as a negative control. Fits and EC50 (concentrations giving 50% of OD at saturation) were determined with GraphPad software.

Competitive ELISA with F5-IgG

One hundred µL of F5-IgG1 solution at 1 mg/mL were combined with 1 µL of a freshly prepared solution of functionalized biotin (EZ Link Sulfo NHS LC Biotin, Thermo Scientific) at 15 µg/µL in water for 30 min, at room temperature (RT). The biotinylation reaction was quenched with 2 µL of Tris buffer pH 7.4 500 mM. Free biotin was removed by filtration with Vivaspin 500 (MWCO 10 kDa) device using PBS as an exchange buffer. Antibody concentration was evaluated as above.

Maxisorp 96-well plates were coated with recombinant LTBP2 and saturated as above. A hundred µL of biotinylated F5-IgG1 (20 nM) combined to non–biotinylated anti-LTBP2 antibodies or rituximab (equimolar or twice more concentrated) in MPBS were added for 2h at RT. Bound biotinylated F5-IgG1 was detected using HRP-conjugated streptavidin (Thermo Scientific) (one hour at RT) followed by the same steps than for the indirect ELISA above. Reduction of the signal obtained with a given antibody indicates competition for binding to LTPB2 of F5-IgG1 and the corresponding antibody and therefore, identical or close binding sites for LTBP2 for both antibodies.

Analysis of Che et al. (2021) CRC-LM single-cell data

A scRNAseq public dataset of 6 CRC-LM was analyzed (Che et al., 2021). Cells coming from CRC primary tumors and peripheral blood, or with less than 1000 detected genes were discarded. The 23,576 remaining single-cell transcriptomes were then normalized and log-transformed as our data. After discarding genes detected in less than 1% of the cells, the 5,000 most variable genes (coefficient of variation) among the 10,000 most expressed genes (average expression) were selected for PCA. The 30 first principal component were submitted to tSNE projection (perplexity = 30). Hierarchical clustering was performed as for our data on the 5,000 genes submitted to PCA. The clustering revealed one group of 329 cells containing both endothelial cells and CAFs (data not shown). CAFs were isolated by performing a second hierarchical clustering based on a set of endothelial cells markers (*PECAM1*, *CDH5*, *CLDN5*, *EGFL7*, *FLT1*) and CAFs markers (*ACTA2*, *PDGFRB*, *COL1A1*, *BGN*, *COL1A2*) (data not shown). A total of 258 CAFs were identified accordingly. We next clustered these 258 CAFs based on the 180 genes of our 6 signatures of 30 genes each: ECM-CAFS, Ctr-CAFs, CP-CAFs, CS-CAFs, Ctr-CAF-I, and Ctr-CAF-II.

Supplementary Results

Supplementary Tables

Supplementary Table 1. Demographic data.

Patient	Sequenced tumors	Gender	Age	Primary tumor location	Mutations	MSI	Last treatment	Size (cm x cm)
P1	One metastasis	F	71	Sigmoid	-	No	FOLFIRI+Cetuximab/Irinotecan+Cetuximab	2,8 x 2,5
P2	One metastasis	F	84	High rectum	-	No	FOLFOX	1,8 x 1,4
P3	One metastasis	M	69	Inferior rectum	-	No	FOLFOX	3,4 x 2,4
P4	Two metastases	F	64	Recto-sigmoid hinge	-	No	FOLFOX+Cetuximab/Xeloda+ Cetuximab	2,8 x 2,2 (A)<br>1,9 x 1,9 (B)
P5	One metastasis	M	82	Right colon	KRAS	No	FOLFOX	3 x 2

Supplementary Table 2. Cell numbers.

	Metastases					
	P1_MP	P2_MP	P3_MP	P4_MPa	P4_MPb	P5_MP
**CAFs**	26	28	622	1,587	1,533	601
**EPCAM+**	801	655	1549	848	1024	454

Supplementary Table 3. CAF subpopulation signature genes.

	Ctr-CAF-I	Ctr-CAF-II	CS-CAFs	CP-CAFs	Ctr-CAFs	ECM-CAFs
1	CLU	RGS5	APOD	POSTN	MCAM	MMP2
2	SNCG	PI15	C3	MMP11	PTP4A3	ITGBL1
3	C12orf75	HIGD1B	CFD	COL10A1	APOLD1	COMP
4	MT1A	OLFML2A	FBLN1	COL11A1	CPM	GREM1
5	SORBS2	NDUFA4L2	PTGDS	SLC6A6	GPRC5C	CYP1B1
6	GADD45G	COX4I2	PTX3	NTM	ADAMTS9	CTHRC1
7	ADIRF	GJC1	C7	ARL4C	SPARCL1	LTBP2
8	MT1X	GJA4	ITM2A	PLAU	CSRP2	COL8A1
9	PDK4	ADAP2	IGF1	COL1A1	TINAGL1	PDGFRA
10	RERGL	KLHL23	SFRP2	FNDC1	ITGA7	IGFBP3
11	PLN	GPR4	ABI3BP	SULF1	ADAMTS4	VCAN
12	MT1M	TMEM74B	COLEC12	INHBA	CSPG4	ISLR
13	KCNMA1	NRARP	SERPINF1	WNT5A	CRIP1	LUM
14	NTRK3	EDNRB	SCARA5	NPR3	PGF	THBS2
15	SBSPON	PLCE1	SCN7A	SUGCT	LRRC10B	PDPN
16	ITIH3	TPPP3	TNXB	ITGA2	GPR20	COL6A3
17	ANGPTL1	SSTR2	SRPX	COL5A1	SERPINI1	EFEMP1
18	MYH11	KCNJ8	SFRP4	ITGA11	CLMN	FBLN2
19	SPEG	PLA2G5	FGL2	HOPX	FABP4	HTRA3
20	ITGA8	PLXDC1	RARRES1	COL3A1	SYNM	LXN
21	KLF2	ENPEP	OMD	HHIP	SLC7A2	PTGIS
22	BCAM	EGFL6	OGN	PLPP4	RASD1	MOXD1
23	ID1	SEMA5B	CCDC80	ADAMTS2	LDB3	MMP23B
24	GLDN	FAM162B	CHRDL1	UNC5B	ATP1A2	FAP
25	AC092164.1	TRPC6	BOC	CDH2	ESAM	PDGFD
26	CASQ2	FAM13C	DPT	PDGFC	LGI4	MEG3
27	C2orf40	CAMK2N1	FBLN5	TYROBP	KCNA5	LOXL1
28	BRSK2	TBX2	GPRC5A	PTK7	AC100803.3	PODN
29	DES	IMPA2	C16orf89	F5	CPE	COL1A2
30	ACTG2	ARHGAP29	ABCA8	COL5A2	EPAS1	CST2

Supplementary Table 4. Intersection between pan-cancer CAF populations[3] and CRC-LM CAF population signature genes. The authors indicated that C7 to C11 clusters represent commonly found CAFs. C1 to C6 are tissue-specific (colon and ovary). (P-values obtained with a hypergeometric test.)

	Ctr-CAF-I	Ctr-CAF-II	CS-CAFs	CP-CAFs	Ctr-CAFs	ECM-CAFs
**C1_KCNN3**			**P=9.6e-13** (DPT, C7, SCN7A, OGN, SRPX, FBLN1)	**P=3.4e-2** (FNDC1)	**P=3.4e-2** (SPARCL1)	**P=5.6e-4** (PDGFRA, LUM)
**C2_ADAMDEC1**			**P=5.3e-6** (CFD, ABCA8, SCARA5)			
**C3_SOX6**	**P=3.4e-2** (GADD45G)	**P=3.4e-2** (EDNRB)		**P=5.6-4** (POSTN, WNT5A)		**P=5.6e-4** (PDGFRA, PDGFD)
**C4_STAR_NF**						
**C5_STAR_CAF**			**P=3.3e-6** (ITM2A)			**P=3.3e-2** (MEG3)
**C6_CALB2**	**P=3.4e-2** (SBSPON)		**P=5.6e-4** (RARRES1, C3)			
**C7_MYH11**	**P=0.0** (MYH11, ADIRF, PLN, RERGL, ACTG2, BCAM, SNCG, SORBS2, C2orf40, C12orf75, NTRK3, CASQ2)				**P=2.6e-10** (CSRP2, TINAGL1, MCAM, PTP4A3, KCNA5)	
**C8_RGS5**		**P=0.0** (RGS5, NDUFA4L2, HIGD1B, COX4I2, GJA4, GJC1, KCNJ8, FAM162B,PLXDC1, ADAP2)			**P=3.4e-8** (MCAM, TINAGL1, ESAM, APOLD1)	
**C9_CFD**	**P=3.5e-2** (CLU)		**P=0.0** (CFD, APOD, FBLN1, TNXB, SFRP2, SRPX, FBLN5, DPT, CCDC80, ITM2A, SERPINF1)			**P=6.2e-2** (FBLN2, EFEMP1, LUM)
**C10_COMP**			**P=5.9e-4** (SFRP4, SFRP2)	**P=0.0** (MMP11, COL1A1, POSTN, COL3A1, COL11A1, COL5A2, INHBA, COL10A1, COL5A1, SULF1, PLAU, HOPX)		**P=0.0** (CTHRC1, COMP, COL1A2, VCAN, THSB2, FAP, COL6A3, COL8A1)
**C11_SERPINE1**	**P=3.5e-2** (DES)	**P=3.5e-2** (EGFL6)	**P=2.6e-10** (C3, RARRES1, IGF1, CCDC80, PTGDS)	**P=5.9e-4** (COL1A1, COL3A1)		**P=6.2e-6** (LXN, PTGIS, COL1A2)

Supplementary Table 5. Intersection between breast cancer primary tumor CAF-S1 subpopulations[4] and CRC-LM CAF population signature genes. (P-values obtained with a hypergeometric test.)

	**Ctr-CAF-I**	**Ctr-CAF-II**	**CS-CAFs**	**CP-CAFs**	**Ctr-CAFs**	**ECM-CAFs**
**S1 (0) (ecm myCAF)**			**P = 5.9e-4** (SFRP2, CCDC80)	**P = 1.1e-12** (COL10A1, COL11A1, FNDC1, SULF1, COL3A1, PLPP4)		**P = 4.0e-15** (ITGBL1, COMP, COL8A1, ISLR, COL6A3, MMP23B, LOXL1)
**S1 (1) (detox iCAF)**			**P = 2.2e-16** (PTX3, C7, TNXB, OGN, CHRDL1, FBLN5, C16orf89)			
**S1 (2) (IL iCAF)**	**P = 2.9e-2** (GADD45G)		**P = 9.4e-11** (C7, ITM2A, COLEC12, SCARA5, FBLN5)			**P = 4.0e-4** (PDGFD, MEG3)
**S1 (3) (TGFB myCAF)**				**P = 2.9e-4** (COL10A1, COL3A1)		**P = 3.9e-11** (COMP, COL6A3, HTRA3, LOXL1, CST2)
**S1(4) (wound myCAF)**			**P = 1.1e-15** (IGF1, SFRP2, SFRP4, OMD, OGN, CCDC80, DPT)	**P = 4.2e-4** (COL11A1, COL3A1)		**P = 1.1e-10** (ITGBL1, COMP, COL8A1, FBLN2, MMP23B)
**S1 (5) (IFN iCAF)**		**P = 2.0e-2** (PLXDC1)	**P = 9.8e-7** (C7, ABI3BP, COLEC12)			**P = 2.0e-2** (CYP1B1)
**S1 (all)**			**P = 5.3e-6** (PTX3, SFRP2, ABI3BP, SCARA5, SFRP4, FGL2, OMD, OGN, CCDC80, CHRDL1, DPT, FBLN5)	**P = 1.4e-6** (COL10A1, COL11A1, FNDC1, COL3A1)		**P = 1.4e-14** (COMP, GREM1, PDGFRA, ISLR, COL6A3, HTRA3, PODN, CST2)

Supplementary Table 6. Healthy and cirrhotic liver mesenchymal cell signature genes.

	VSMC	HSC	SAMes
1	PLN	IGLC2	LUM
2	SORBS2	HIGD1B	PTGDS
3	PHLDA2	IGHA2	IGFBP3
4	SNCG	RGS5	COL1A1
5	MT1M	TM4SF1	COL3A1
6	RP5-966M1.6	FABP5	DPT
7	MYH11	PLAT	COL1A2
8	ACTG2	FABP4	DCN
9	ADIRF	AGT	FBLN1
10	CRIP1	CPE	CCL19
11	ITGA8	NPR3	C3
12	PDGFA	IGHA1	C7
13	SBSPON	SSTR2	SERPINF1
14	PPP1CB	FCN3	TIMP1
15	AC097724.3	AC018647.3	CXCL12
16	KCNMB1	IMPA2	LXN

Supplementary Table 7. Intersection between healthy and cirrhotic mesenchymal cell signature genes[5] and CRC-LM CAF subpopulation signature genes. (P-values obtained with a hypergeometric test.)

		CRC-LM CAF subtypes					
		**Ctr-CAF-I**	**Ctr-CAF-II**	**CS-CAFs**	**CP-CAFs**	**Ctr-CAFs**	**ECM-CAFs**
Liver mesenchymal cell populations	**VSMC**	**P = 0.0** (PLN, SORBS2, SNCG, MT1M, MYH11, ACTG2, ADIRF, ITGA8, SBSPON, AC097724.1)				**P = 1.4e-2** (CRIP1)	
	**HSC**		**P = 9.4e-10** (HIGDB, RGS5, SSTR2, IMPA2)		**P = 1.4e-2** (NPR3)	**P = 9.2e-5** (CPE, FABP4)	
	**SAMes**			**P = 2.4e-15** (C3, C7, FBLN1, PTGDS, SERPINF1, DPT)	**P = 9.2e-5** (COL1A1, COL3A1)		**P = 9.4e-10** (LUM, IGFBP3, COL1A2, LXN)

Supplementary Table 8. Performance of tested automatic classifiers.

Precision

	VSMCs				HSCs				SAMes			
	**mean**	**Sd**	**min**	**max**	**mean**	**sd**	**min**	**max**	**mean**	**sd**	**min**	**max**
**SVM**	0.90	0.02	0.86	0.95	0.84	0.04	0.79	0.89	0.96	0.04	0.86	1.00
**KNN**	0.89	0.03	0.84	0.96	0.83	0.03	0.76	0.90	***0.97***	0.04	0.86	1.00
**RF**	***0.91***	0.03	0.84	0.97	***0.87***	0.04	0.80	0.93	0.95	0.03	0.84	1.00

Recall

	VSMCs				HSCs				SAMes			
	**Mean**	**Sd**	**min**	**max**	**mean**	**sd**	**min**	**max**	**Mean**	**sd**	**min**	**max**
**SVM**	0.88	0.03	0.84	0.94	0.86	0.02	0.83	0.92	0.93	0.04	0.86	1.00
**KNN**	0.86	0.03	0.80	0.90	0.86	0.03	0.82	0.95	***0.99***	0.02	0.94	1.00
**RF**	***0.90***	0.03	0.85	0.94	***0.88***	0.04	0.80	0.93	0.97	0.03	0.91	1.00

Supplementary Table 9. Deregulated GO Biological Process terms upon siLTBP2

GO ID	Description	FDR
GO:0030335	positive regulation of cell migration	9.22E-05
GO:0031581	hemidesmosome assembly	9.22E-05
GO:0030595	leukocyte chemotaxis	4.16E-04
GO:0030198	extracellular matrix organization	2.37E-03
GO:0071526	semaphorin-plexin signaling pathway	2.37E-03
GO:0032967	positive regulation of collagen biosynthetic process	3.68E-03
GO:0001657	ureteric bud development	3.87E-03
GO:0006954	inflammatory response	4.09E-03
GO:0051281	positive regulation of release of sequestered calcium ion into cytosol	5.98E-03
GO:0010818	T cell chemotaxis	6.49E-03
GO:0008406	gonad development	6.49E-03
GO:0001778	plasma membrane repair	8.72E-03
GO:0032914	positive regulation of transforming growth factor beta1 production	1.31E-02
GO:0042327	positive regulation of phosphorylation	1.71E-02
GO:0071300	cellular response to retinoic acid	1.71E-02
GO:0060449	bud elongation involved in lung branching	1.71E-02
GO:0050710	negative regulation of cytokine secretion	1.71E-02
GO:0040037	negative regulation of fibroblast growth factor receptor signaling pathway	1.95E-02
GO:0030322	stabilization of membrane potential	2.37E-02
GO:0045766	positive regulation of angiogenesis	2.44E-02
GO:0002040	sprouting angiogenesis	2.84E-02
GO:0032526	response to retinoic acid	2.84E-02
GO:0050918	positive chemotaxis	2.84E-02
GO:0007567	parturition	2.84E-02
GO:0006690	icosanoid metabolic process	2.84E-02
GO:0030850	prostate gland development	3.47E-02
GO:1903671	negative regulation of sprouting angiogenesis	3.47E-02
GO:0090050	positive regulation of cell migration involved in sprouting angiogenesis	3.70E-02
GO:0045880	positive regulation of smoothened signaling pathway	3.81E-02
GO:0070373	negative regulation of ERK1 and ERK2 cascade	3.81E-02
GO:0001649	osteoblast differentiation	3.81E-02
GO:0007191	adenylate cyclase-activating dopamine receptor signaling pathway	3.81E-02
GO:0048333	mesodermal cell differentiation	3.81E-02
GO:0010564	regulation of cell cycle process	3.81E-02
GO:0009888	tissue development	4.19E-02
GO:0010976	positive regulation of neuron projection development	4.19E-02
GO:0043087	regulation of GTPase activity	4.19E-02
GO:1902287	semaphorin-plexin signaling pathway involved in axon guidance	4.19E-02
GO:0031018	endocrine pancreas development	4.19E-02
GO:0071305	cellular response to vitamin D	4.19E-02
GO:0050777	negative regulation of immune response	4.19E-02
GO:0046007	negative regulation of activated T cell proliferation	4.61E-02
GO:0048843	negative regulation of axon extension involved in axon guidance	4.61E-02
GO:0048661	positive regulation of smooth muscle cell proliferation	4.61E-02
GO:0098742	cell-cell adhesion via plasma-membrane adhesion molecules	4.61E-02
GO:0001525	angiogenesis	4.99E-02

Supplementary Figures

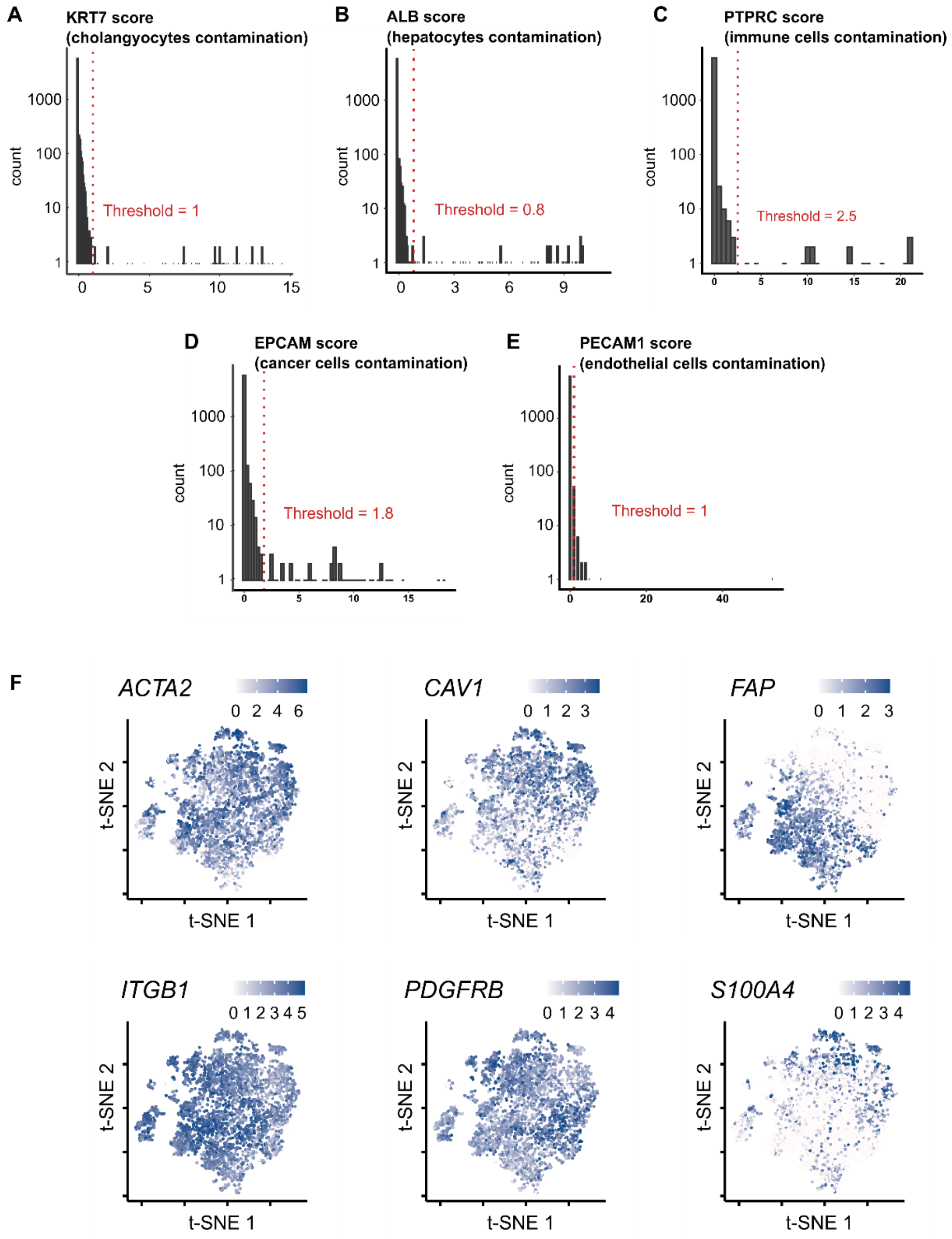

Supplementary Figure 1. (A) Elimination of contaminating cholangiocytes. Ten most *KRT7*-correlated genes: *KRT7*, *C3*, *SERPING1*, *TM4SF4*, *CXCL6*, *BICC1*, *DEFB1*, *DCDC2*, *APCS*, and *SCTR*; correlations between 0.72 and 0.81. Cells with *KRT7*-score ≥ 1 were removed (61 cells). **(B)** Elimination of contaminating hepatocytes. Ten most *ALB*-correlated genes: *ALB*, *AMBP*, *ORM1*, *HP*, *SERPINA1*, *APOA2*, *FGG*, *APOC3*, *APOC1*, *FGB*; correlations between 0.88 and 0.92. Cells with ALB-score ≥ 0.8 were removed (84 cells). **(C)** Elimination of contaminating immune cells. Ten most *PTPRC*(CD45)-correlated genes: *PTPRC*, *LAPTM5*, *CD3D*, *CD3G*, *CD2*, *CD52*, *CD7*, *CD3E*, *CD48*, and *ARHGAP9*; correlations between 0.61 and 0.74. Cells with *PTPRC*-score ≥ 2.5 were removed (17 cells). **(D)** Elimination of contaminating epithelial (cancer) cells. Ten most EPCAM-correlated genes: *EPCAM*, *CEACAM5*, *TFF3*, *CEACAM6*, *PHGR1*, *FXYD3*, *AGR2*, *TSPAN8*, *S100P*, and *LGALS4*; correlations between 0.67 and 0.80. Cells with *EPCAM*-score ≥ 1.8 were removed (48 cells). **(E)** Elimination of contaminating endothelial cells based on CD31 (PECAM1) expression. Cells with *PECAM1* UMI ≥ 1 (non-normalized data) were removed (66 cells). **(F)** Gene expression of 6 classical CAF markers showing different patterns: *ACTA2*, *CAV1*, *ITGB1* and *PDGFRB* were uniformly expressed, while *FAP* and *S100A4* were only expressed in two distinct CAF subsets. This observation is discussed in Results.

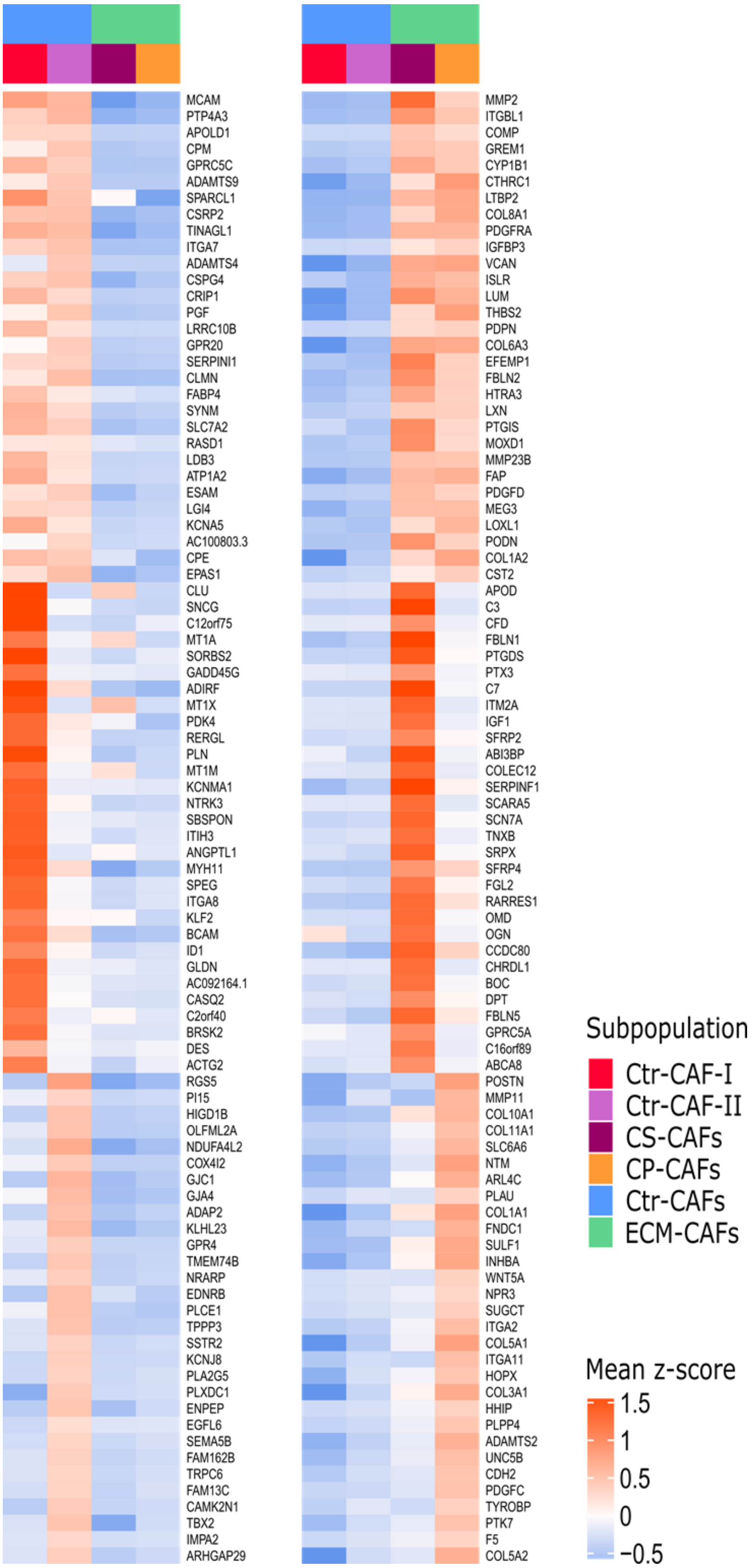

Supplementary Figure 2. CAF subpopulation gene signatures average expression.

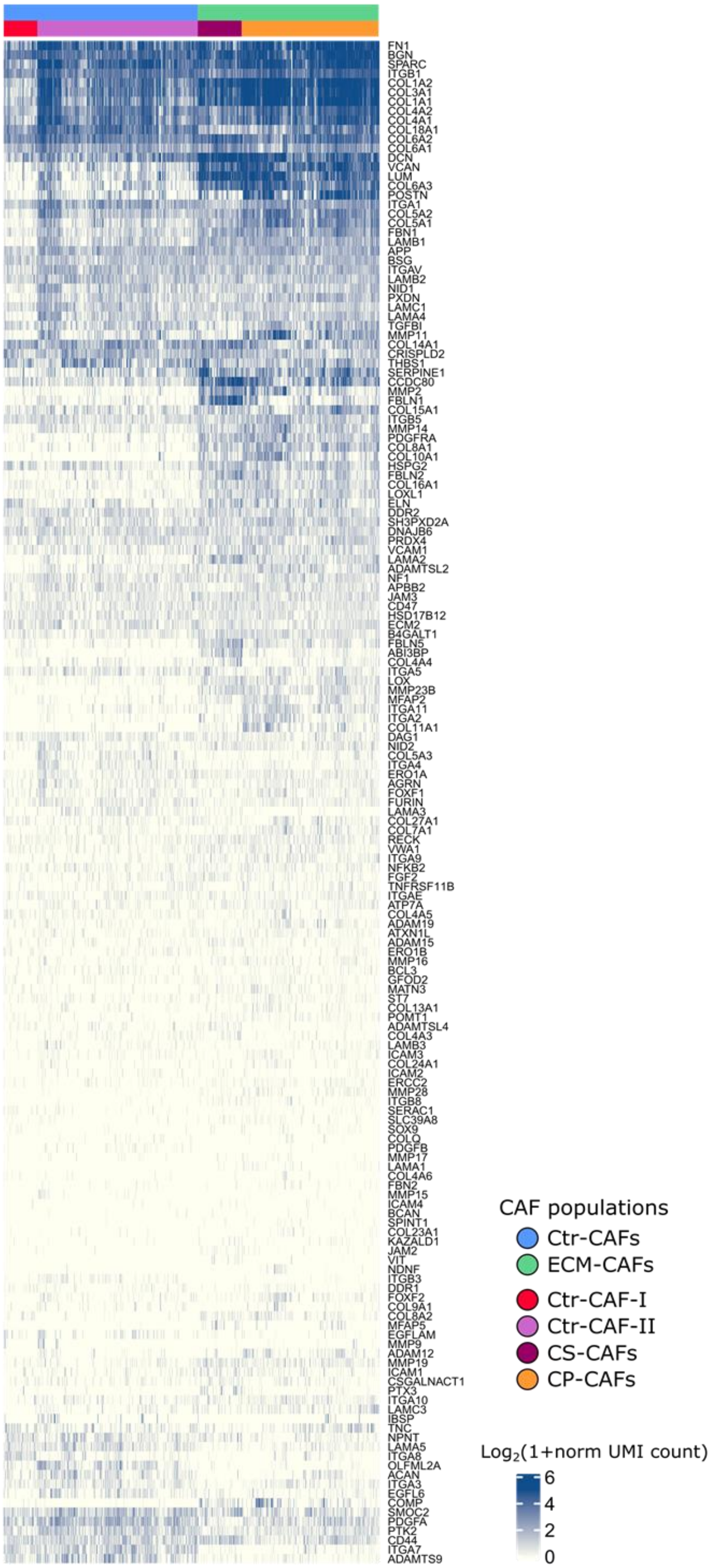

Supplementary Figure 3. ECM remodeling-associated genes and CAF markers.

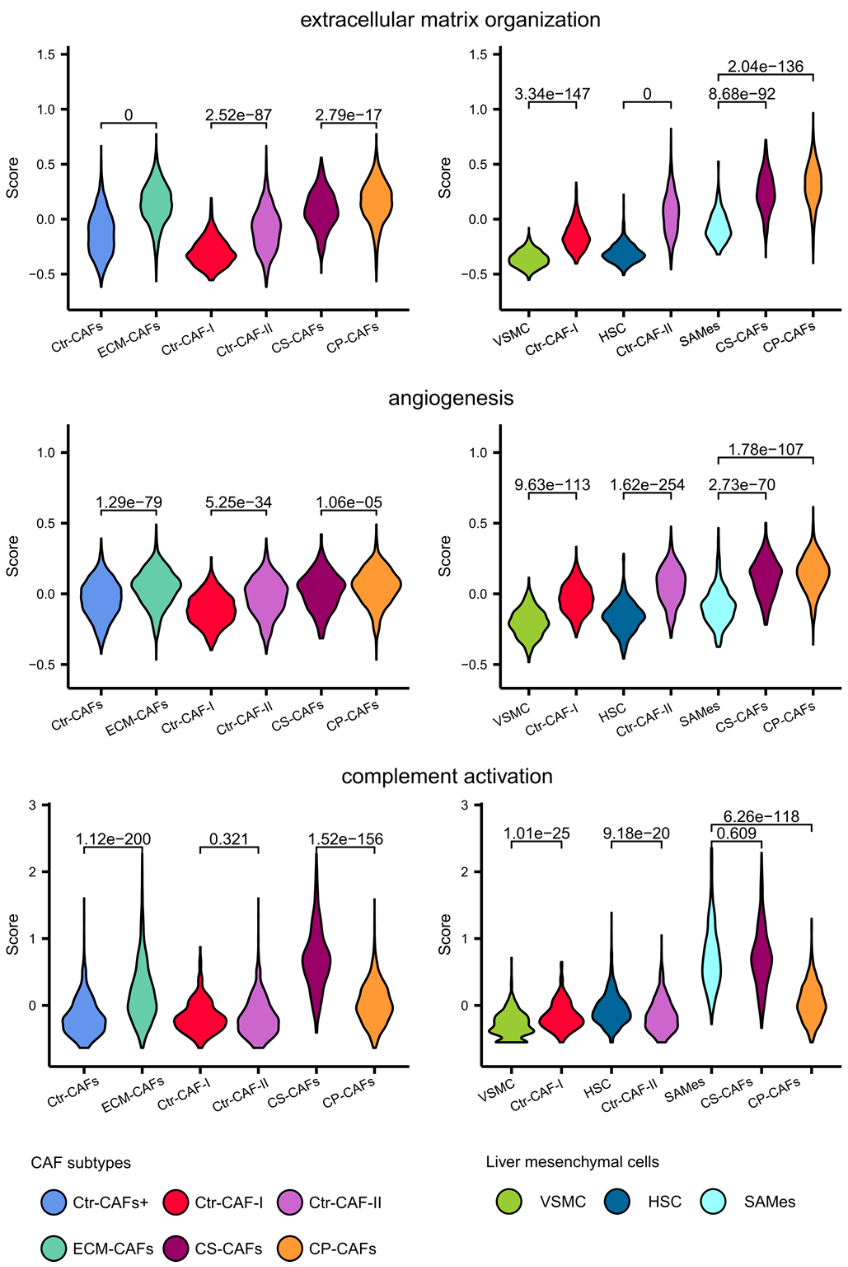

Supplementary Figure 4. GO Biological Process score comparisons (scores are averages of gene z-scores). Left panels compare CRC-LM CAF populations. Right panels compare these populations with their closest phenotypic match among mesenchymal cells in the liver.[5] (Wilcoxon test.)

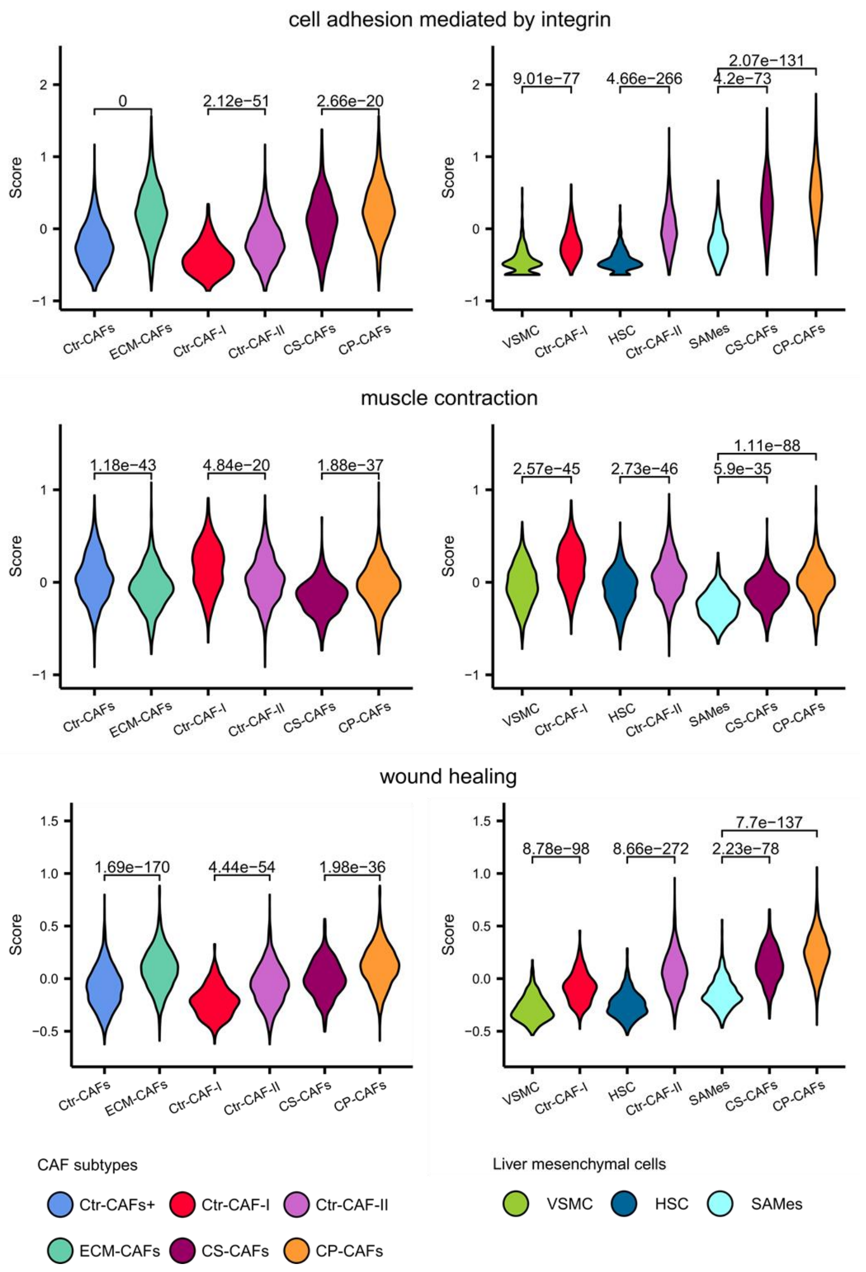

Supplementary Figure 5. GO Biological Process score comparisons (scores are averages of gene z-scores). Left panels compare CRC-LM CAF populations. Right panels compare these populations with their closest phenotypic match among mesenchymal cells in the liver.[5] (Wilcoxon test.)

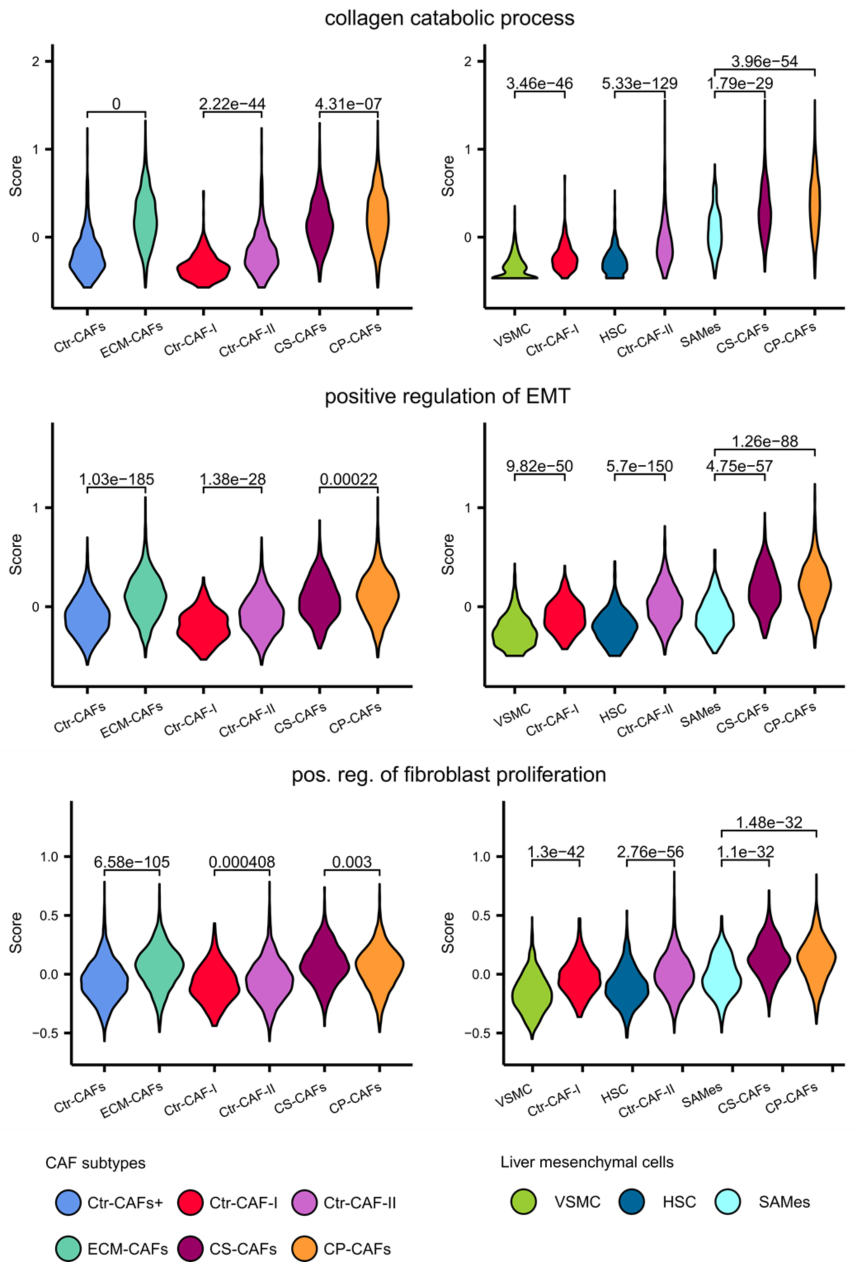

Supplementary Figure 6. GO Biological Process score comparisons (scores are averages of gene z-scores). Left panels compare CRC-LM CAF populations. Right panels compare these populations with their closest phenotypic match among mesenchymal cells in the liver.[5] (Wilcoxon test.)

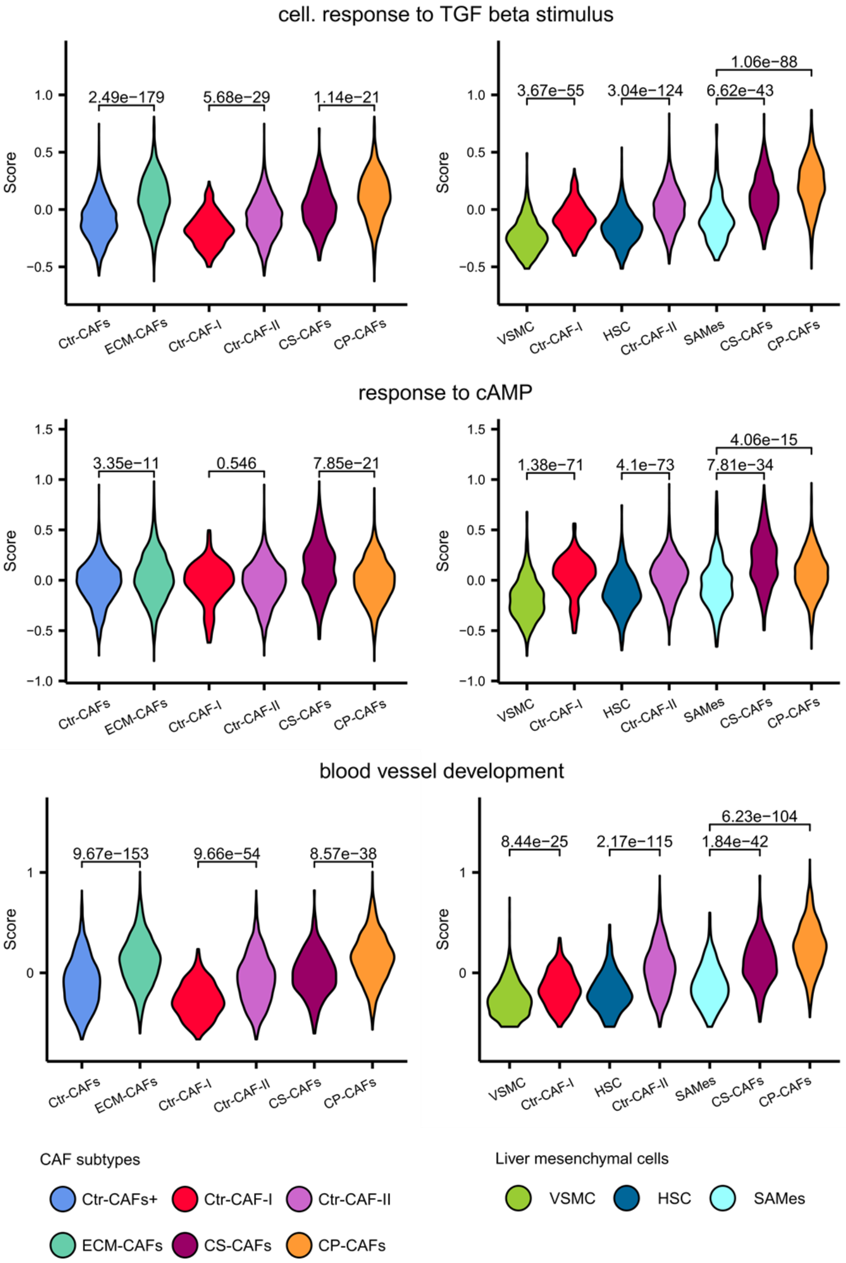

Supplementary Figure 7. GO Biological Process score comparisons (scores are averages of gene z-scores). Left panels compare CRC-LM CAF populations. Right panels compare these populations with their closest phenotypic match among mesenchymal cells in the liver.[5] (Wilcoxon test.)

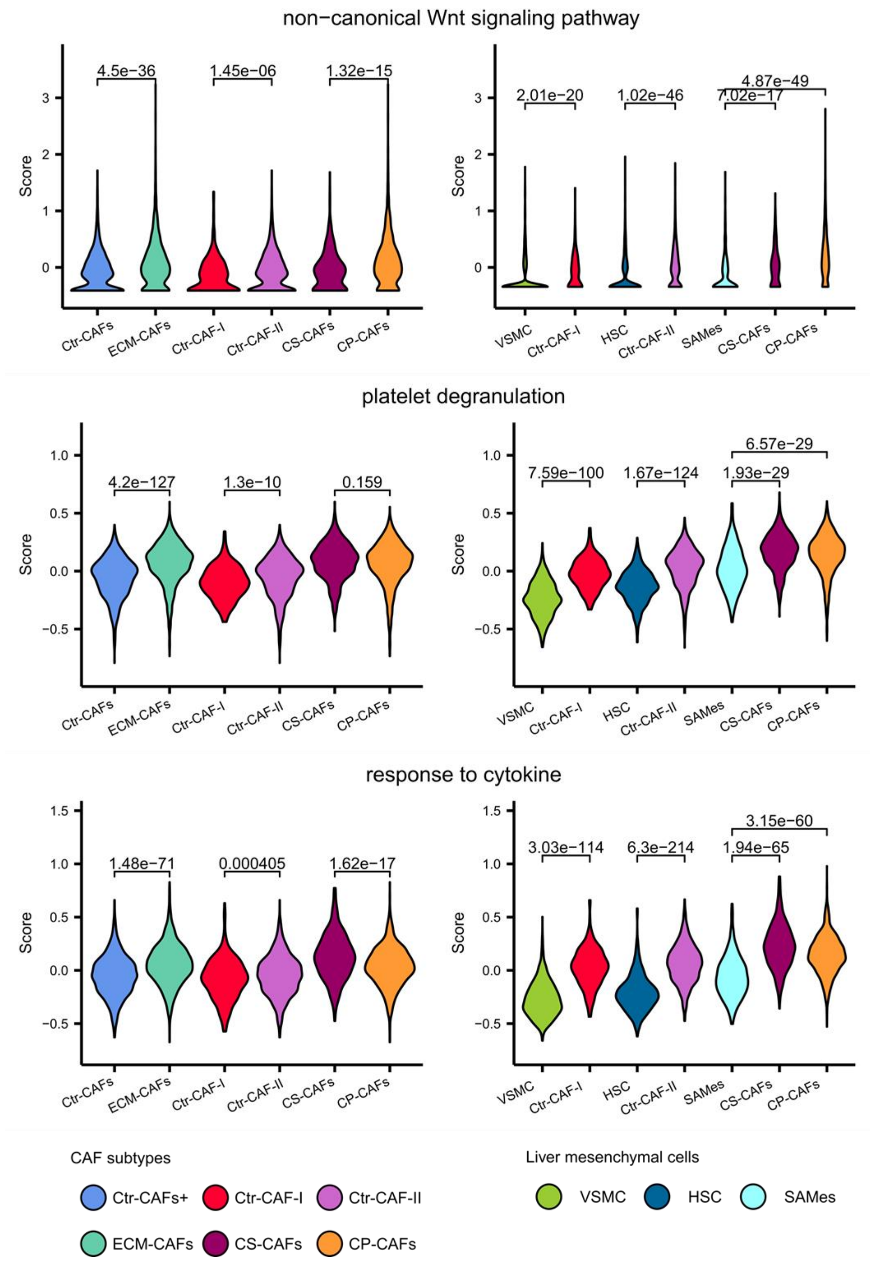

Supplementary Figure 8. GO Biological Process score comparisons (scores are averages of gene z-scores). Left panels compare CRC-LM CAF populations. Right panels compare these populations with their closest phenotypic match among mesenchymal cells in the liver.[5] (Wilcoxon test.)

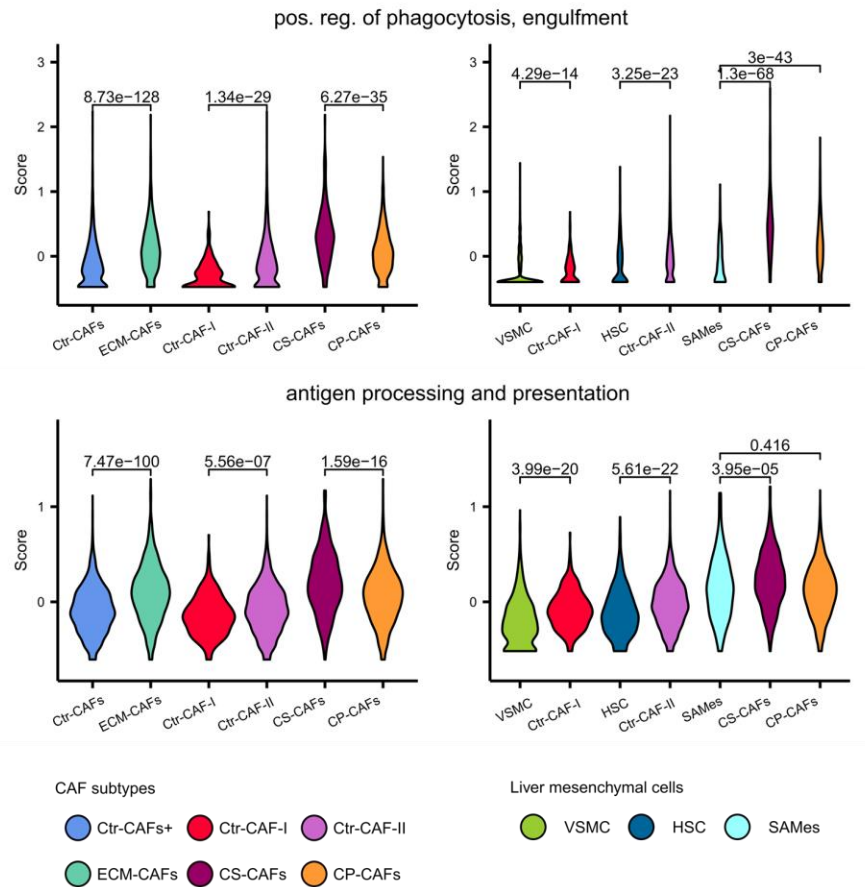

Supplementary Figure 9. GO Biological Process score comparisons (scores are averages of gene z-scores). Left panels compare CRC-LM CAF populations. Right panels compare these populations with their closest phenotypic match among mesenchymal cells in the liver.[5] (Wilcoxon test.)

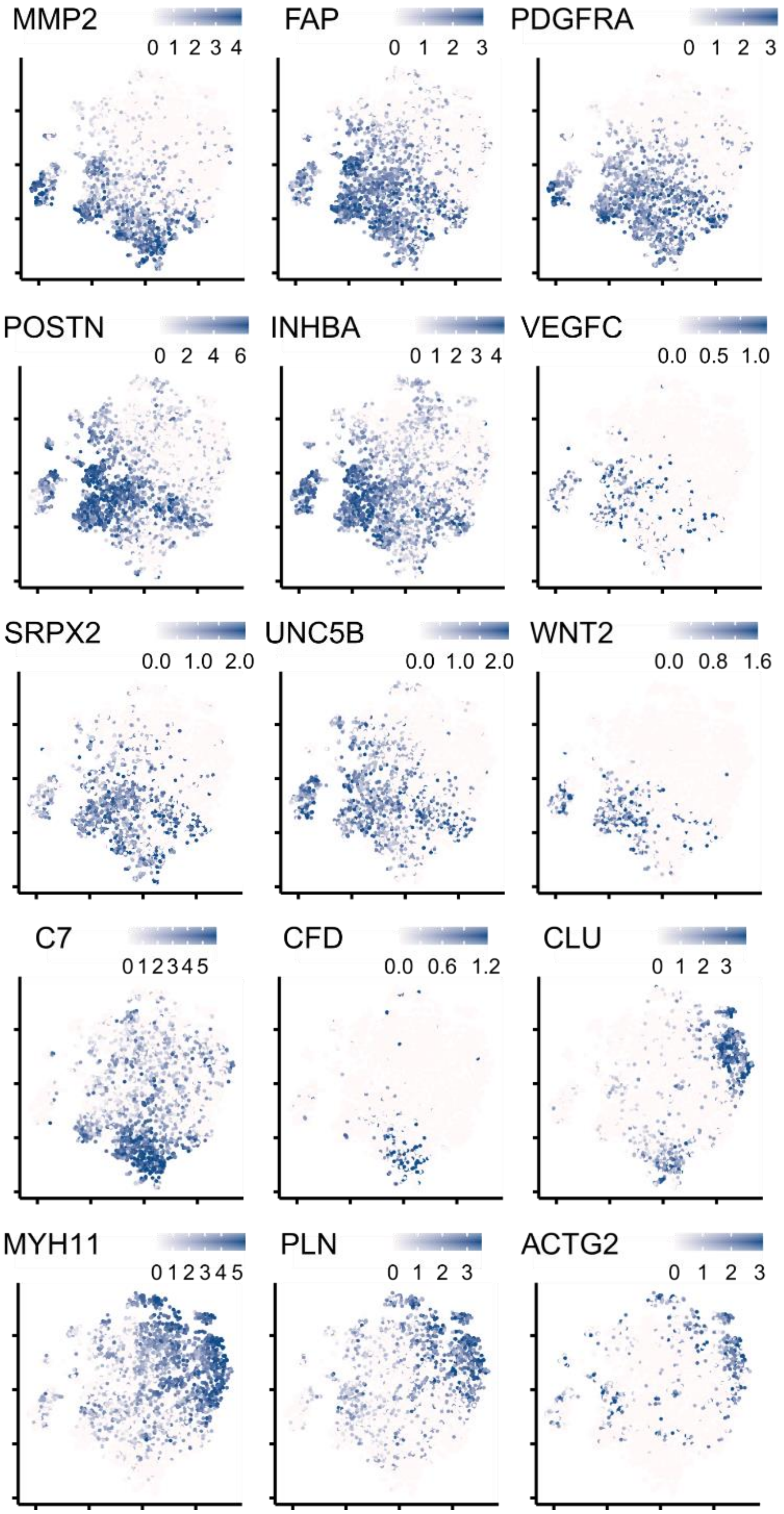

Supplementary Figure 10. Expression of representative genes.

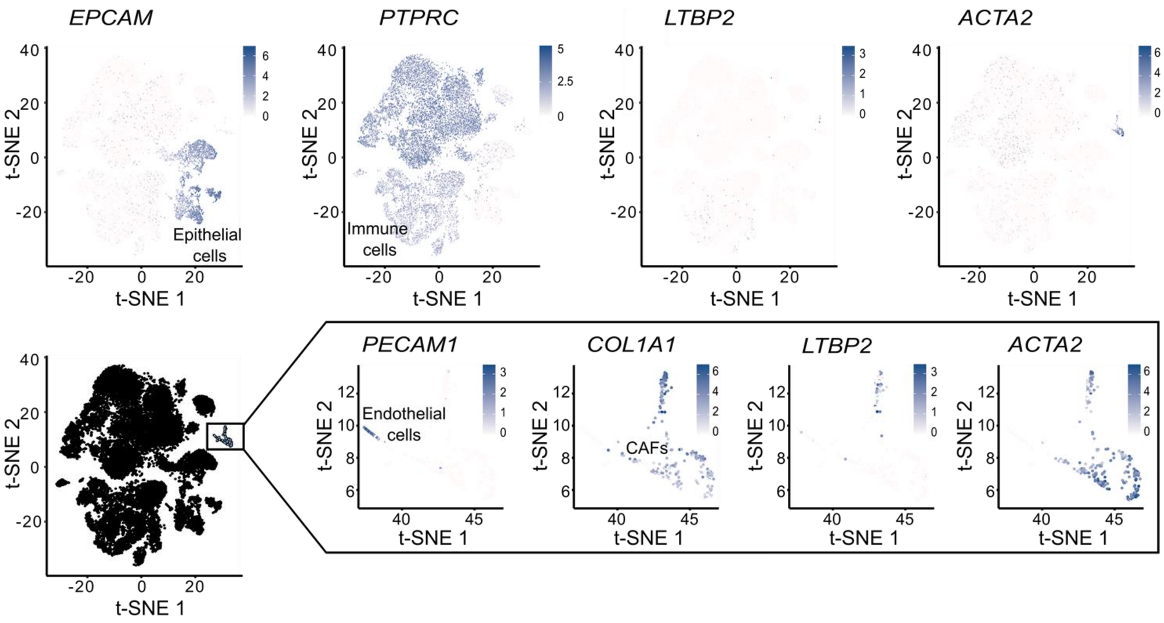

Supplementary Figure 11. Expression of *LTBP2* and *ACTA2* genes in Che et al. Data[6]. We note the absence of expression of the two genes in epithelial and immune cells (top), whereas *ACTA2* (and *COL1A1*) are expressed by all the CAFs and *LTBP2* only by subpopulation of CAFs (bottom). Neither *LTBP2* nor *ACTA2* are expressed by endothelial cells (bottom).

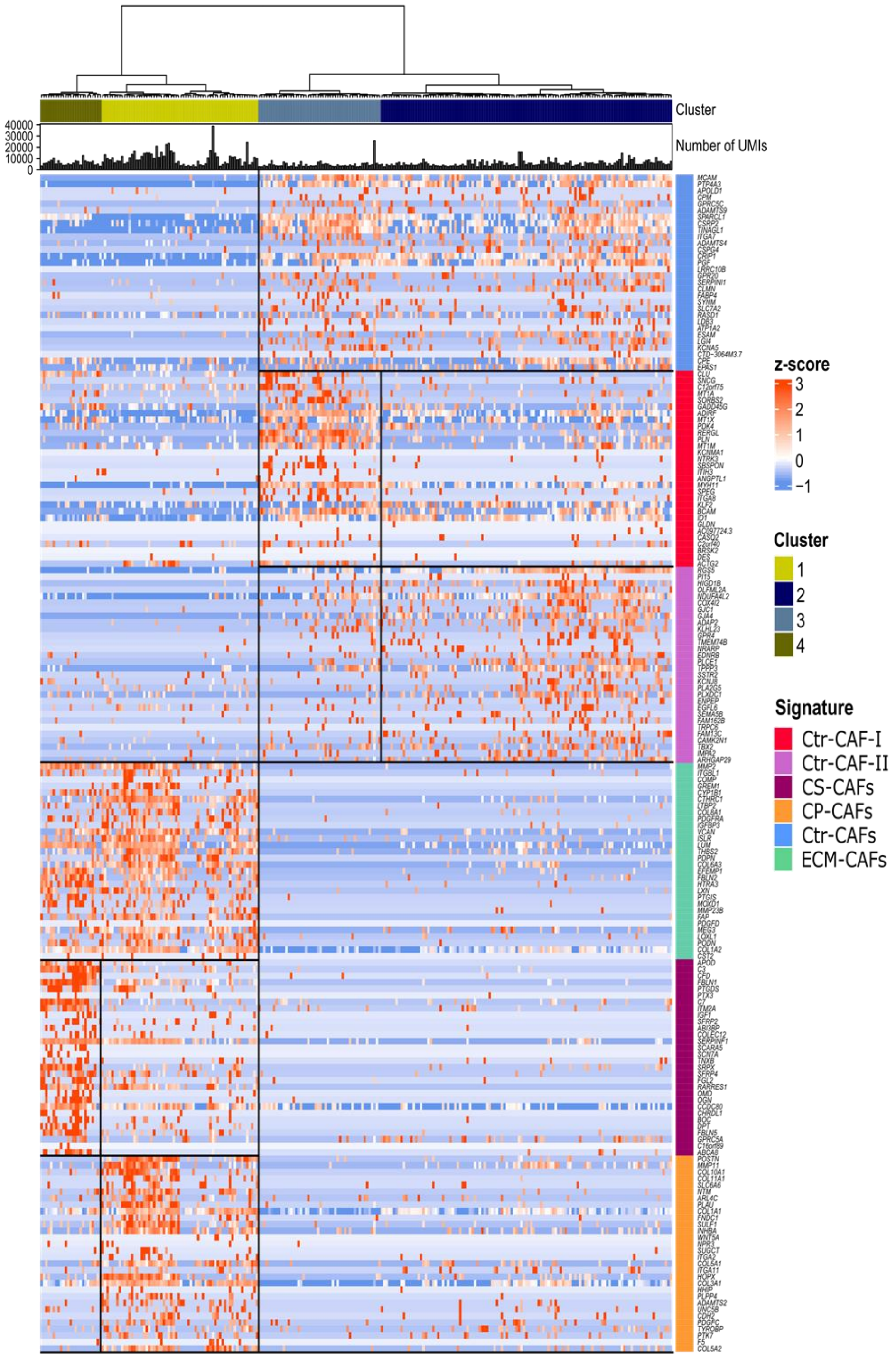

Supplementary Figure 12. Dendrogram and heatmap based on Che et al. CAF transcriptomes[6] restricted to the CAF population-defining signature genes of our study.

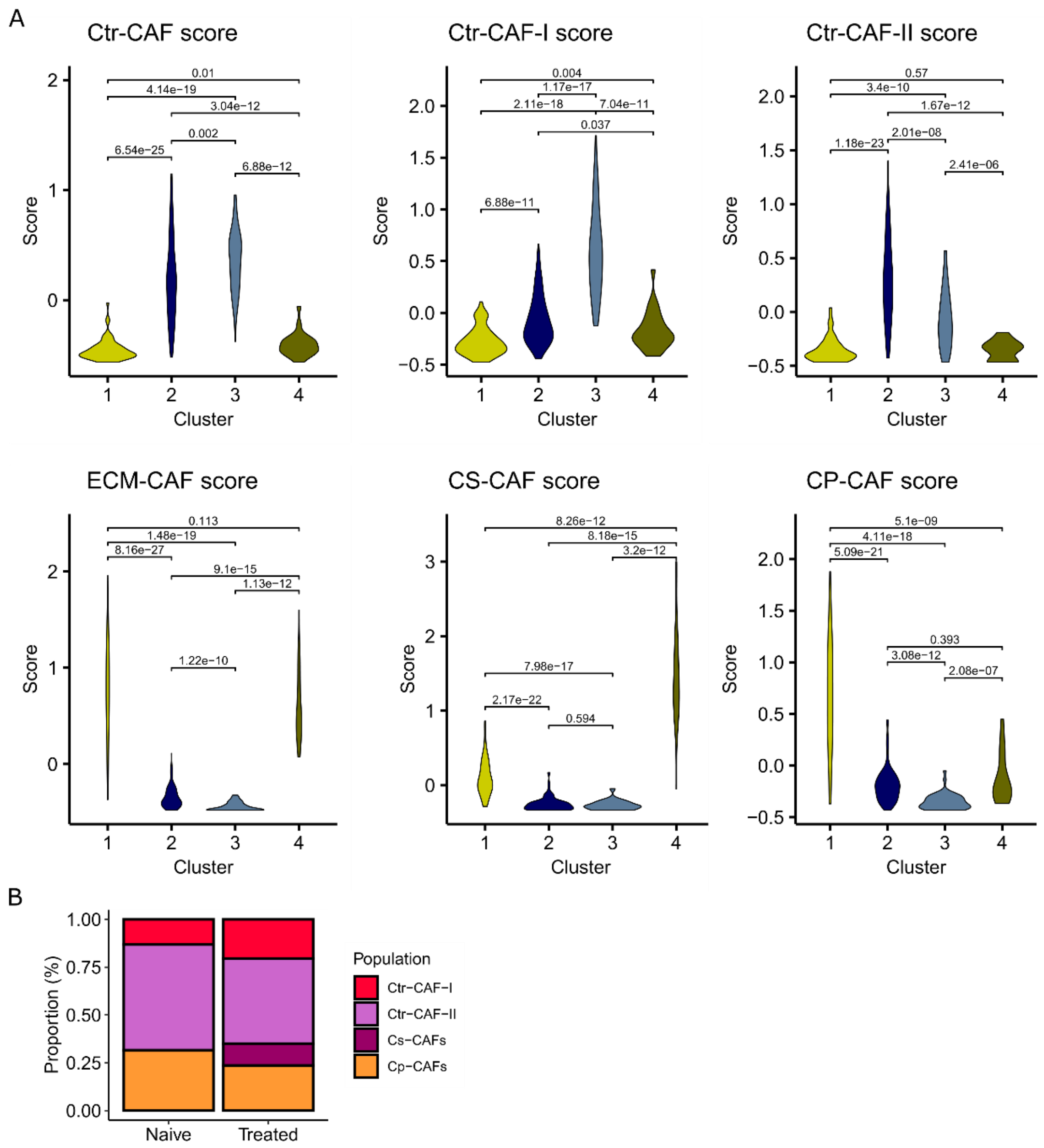

Supplementary Figure 13. (A) Scores of our gene signatures (average of gene z-scores) in the CAFs of Che et al.[6] CAFs were grouped according to the dendrogram of Suppl. Fig. 11 above. (B) Proportion of CAF subtypes before/after chemotherapy in Che et al. No significant difference was found (multinomial test P = 4%, Fisher exact test P = 5%).

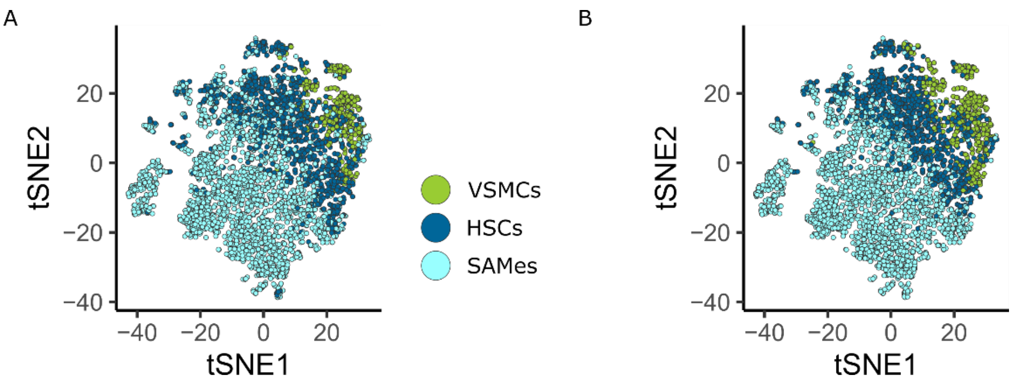

Supplementary Figure 14. (A) Support vector machine inference. (B) K-nearest neighbor inference.

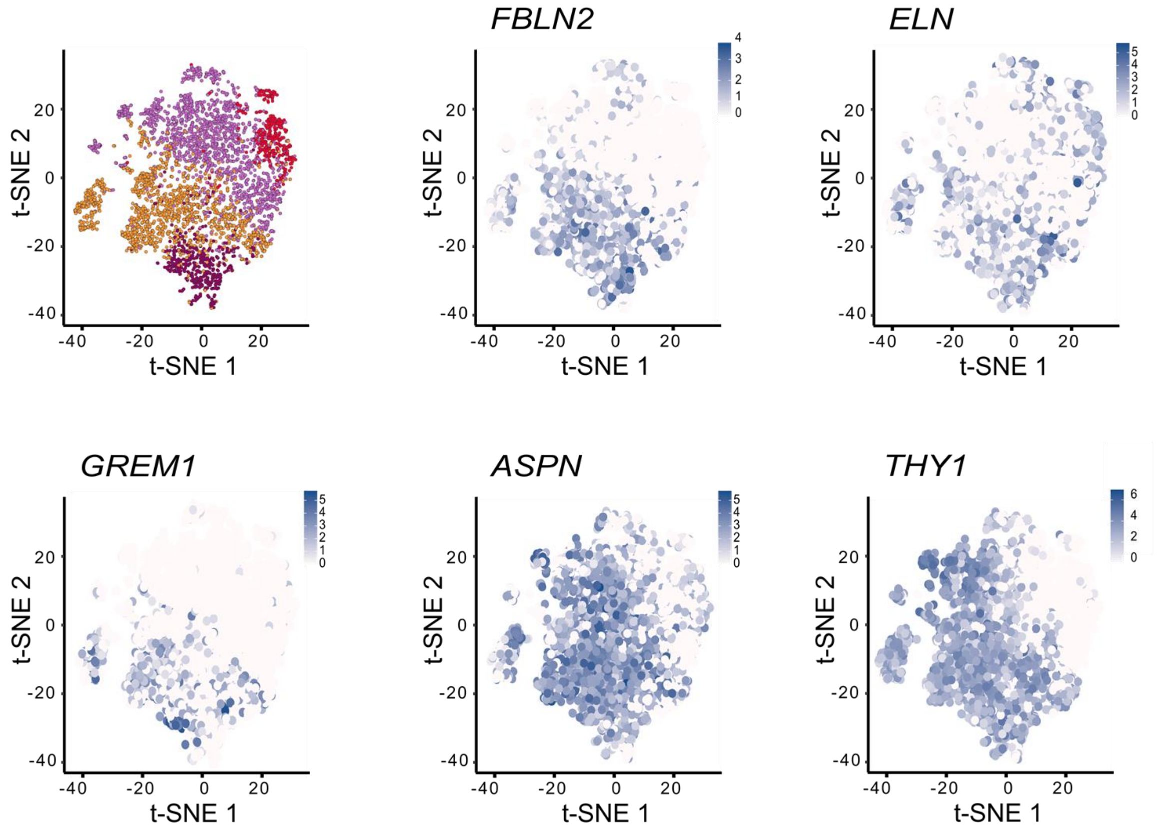

Supplementary Figure 15. Portal fibroblast genes not expressed by HSCs strongly associates with ECM-CAFs.

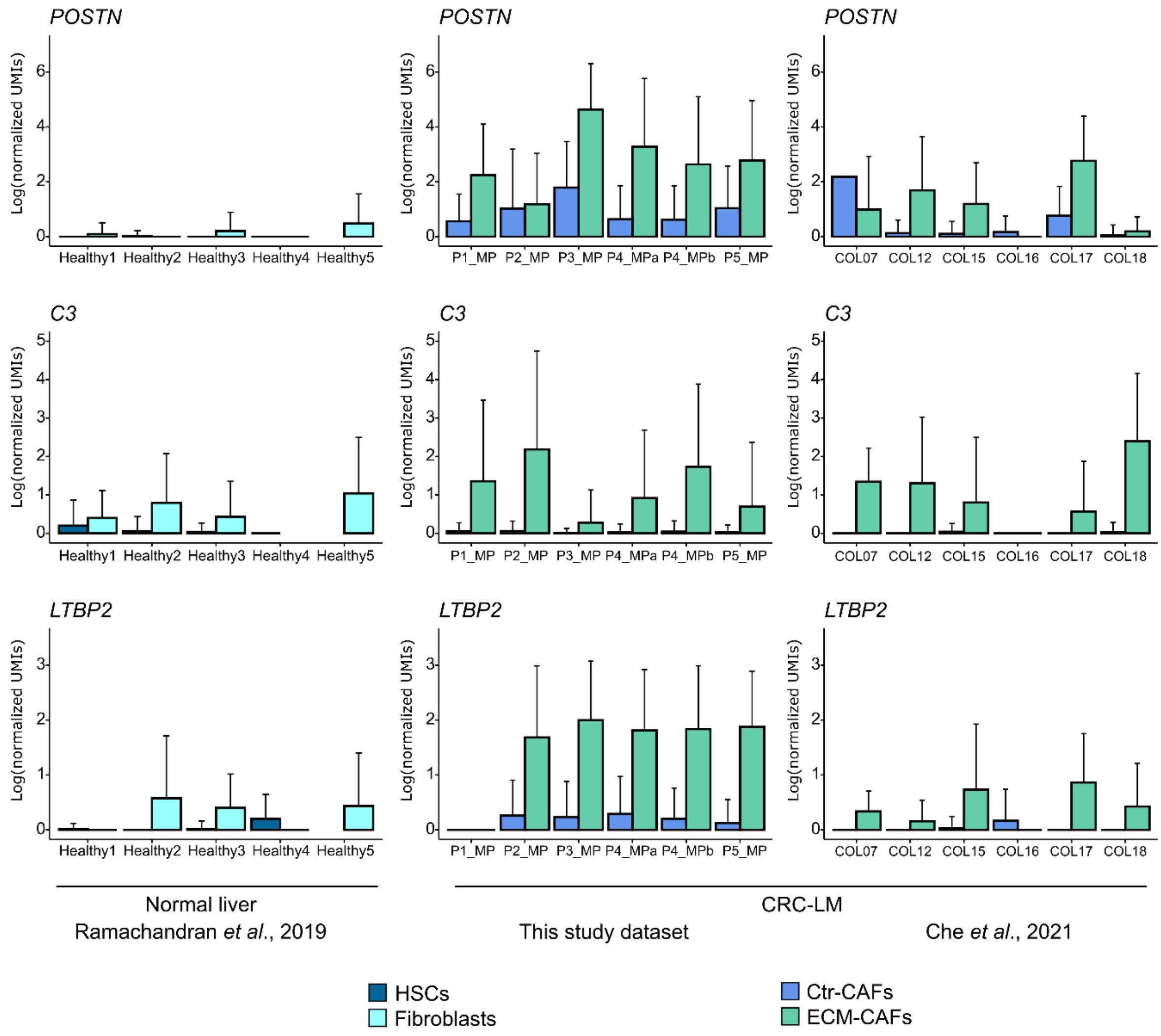

Supplementary Figure 16. *POSTN, C3,* and *LTBP2* expression in 5 healthy liver HSCs and fibroblasts (left), 6 metastases from our 5 patients (center) distinguishing ECM- and Ctr-CAFs, and in Che *et al*. 6 patients (right). Data were normalized by total UMI counts and log transformed according to Materials and Methods of the main paper. Error bars = 1 standard deviations. Bars devoid of error bars contained one cell only (Che *et al*. CAF transcriptomes only covered 258 CAFS in total).

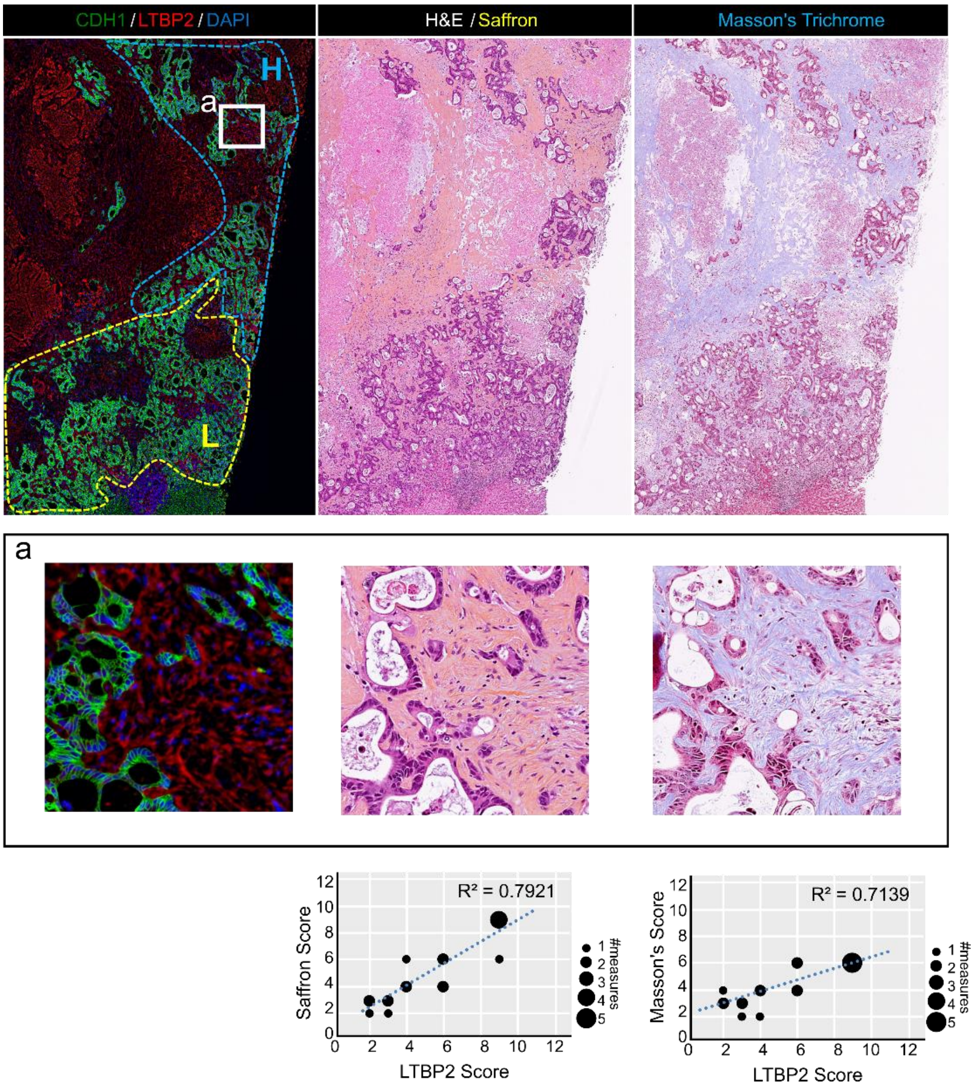

Supplementary Figure 17. Histological analysis of CRC-LM serial sections featuring (from left to right) LTBP2/CADH1 multiplexed fluorescence, hematoxylin/eosin Saffron and Masson's trichrome stain (representative images, DAPI is used as counterstain in IF). Collagen expression was evaluated by both Saffron and Masson's trichrome stains in two distinct areas per section, featuring low (L, yellow region) and high (H, blue region) LTBP2 expressions respectively.

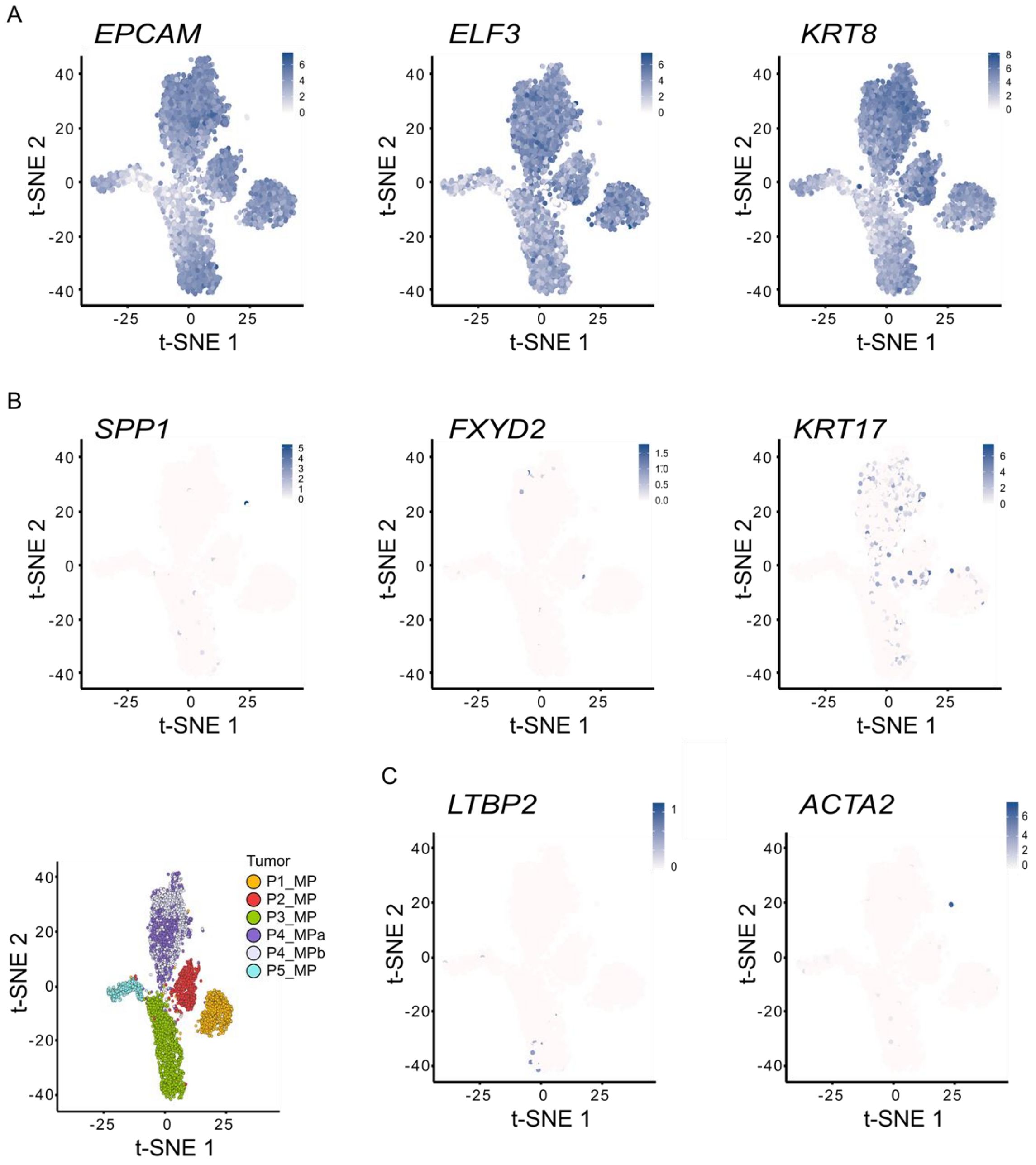

Supplementary Figure 18. (A) Epithelial cancer cell markers as defined in Che at al.[6]. (B) Healthy cholangiocyte markers from PanglaoDB. (C) Absence of ECM- (*LTBP2*) and pan-CAF (*ACTA2*) gene markers in epithelial cancer cells.

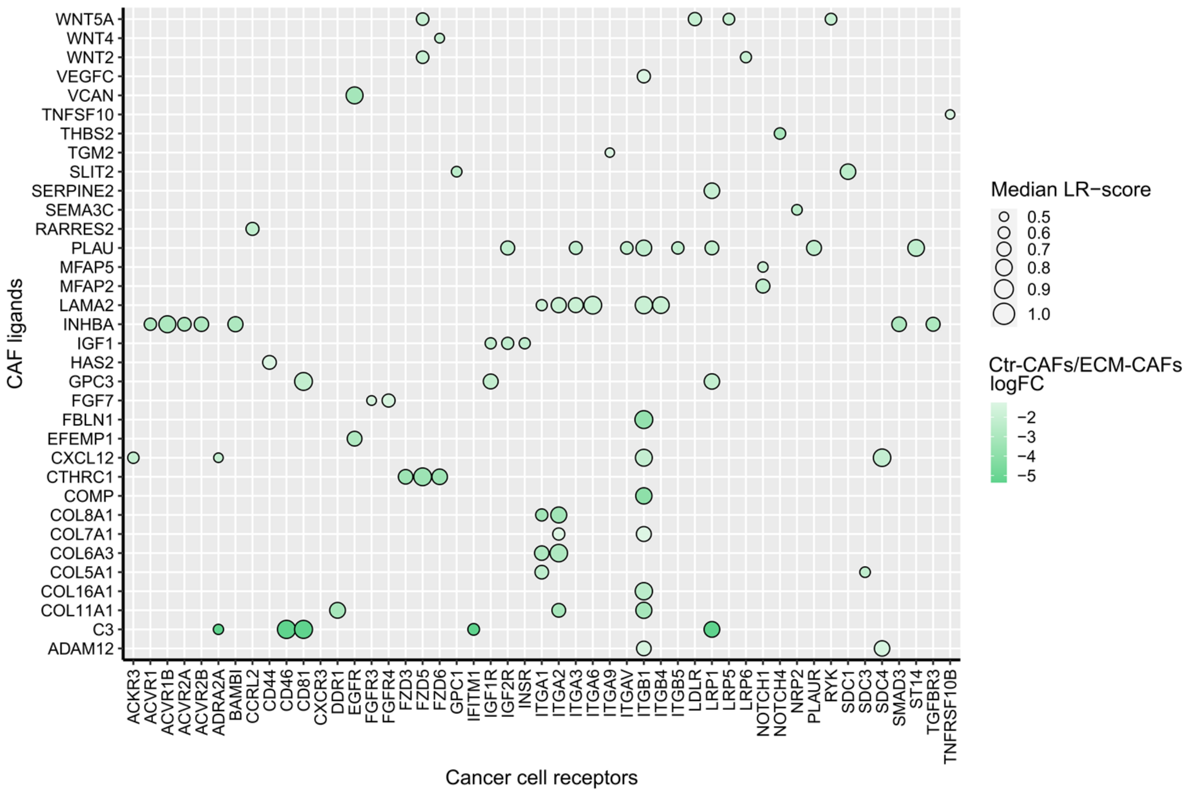

Supplementary Figure 19. Reliable LTBP2+ CAFs to cancer cell LR interactions, featuring a significant increase compared to MCAM+ CAFs.

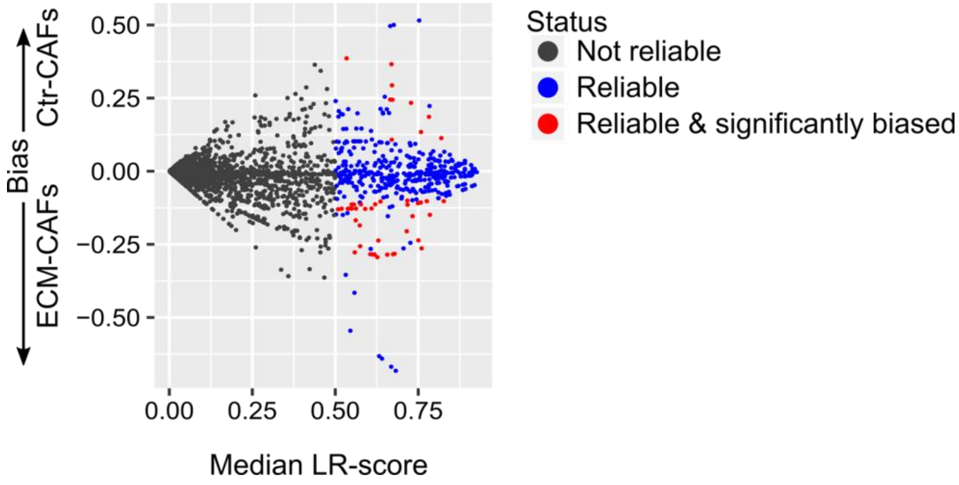

Supplementary Figure 20. Selection of reliable and significantly biased LR interactions between cancer cells and CAFs.

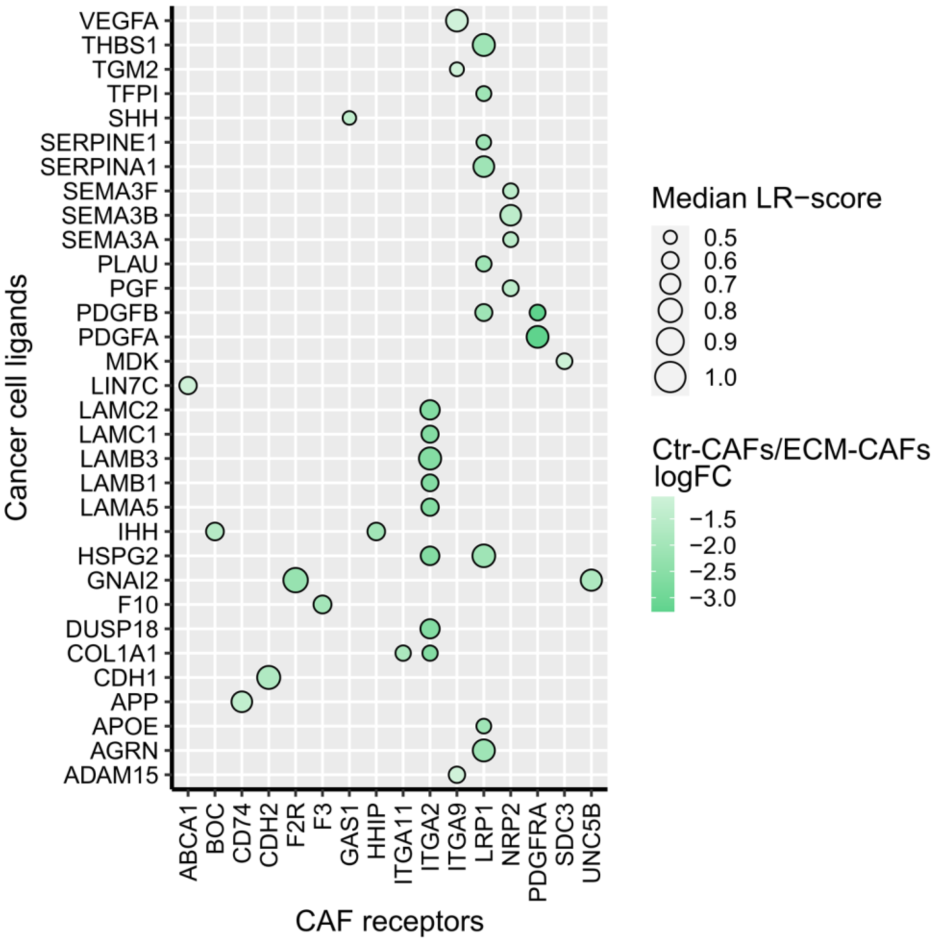

Supplementary Figure 21. Reliable cancer cell to ECM-CAF LR interactions, featuring a significant increase compared to Ctr-CAFs.

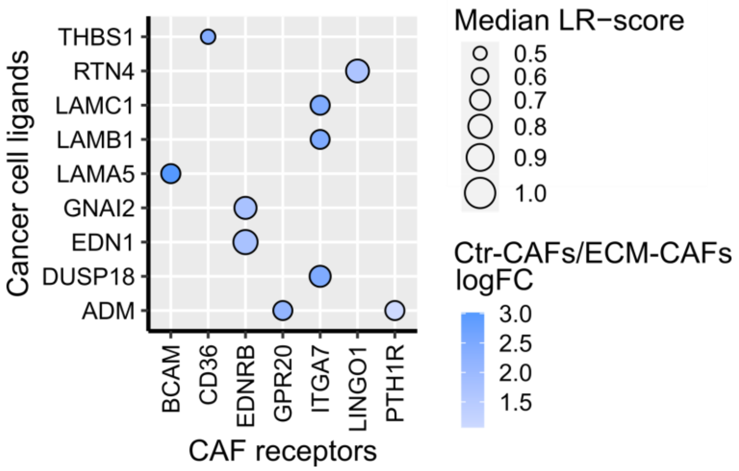

Supplementary Figure 22. Reliable cancer cell to Ctr-CAF LR interactions, featuring a significant increase compared to ECM-CAFs.

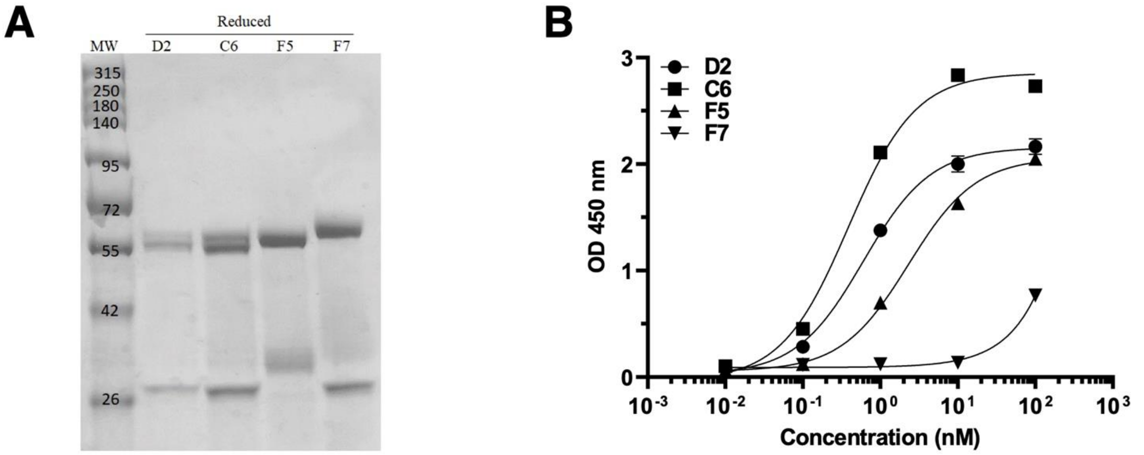

	D2	C6	F5	F7
EC50 (nM)	0.61	0.39	2.3	N/A

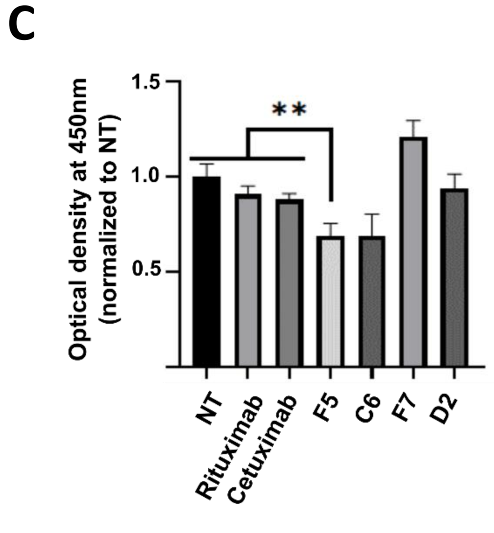

Supplementary Figure 23. Characterization of anti-LTBP2 antibodies. (A) Separative SDS-PAGE of 5 µg of purified anti-LTBP2 IgG1, in reduced conditions. **(B)** Binding of anti-LTBP2 in the IgG1 format to recombinant LTBP2 was assessed by indirect ELISA (see material and methods). EC50 is indicated below the graph. F5-IgG1, C6-IgG1 and D2-IgG1 display EC50 in the nmolar range, EC50 of F7-IgG1 is not deductible from this experiment (n=2). **(C)** Depletion of patient CAFs upon treatment with antibodies against LTBP2. ** P < 0.01, t-test.

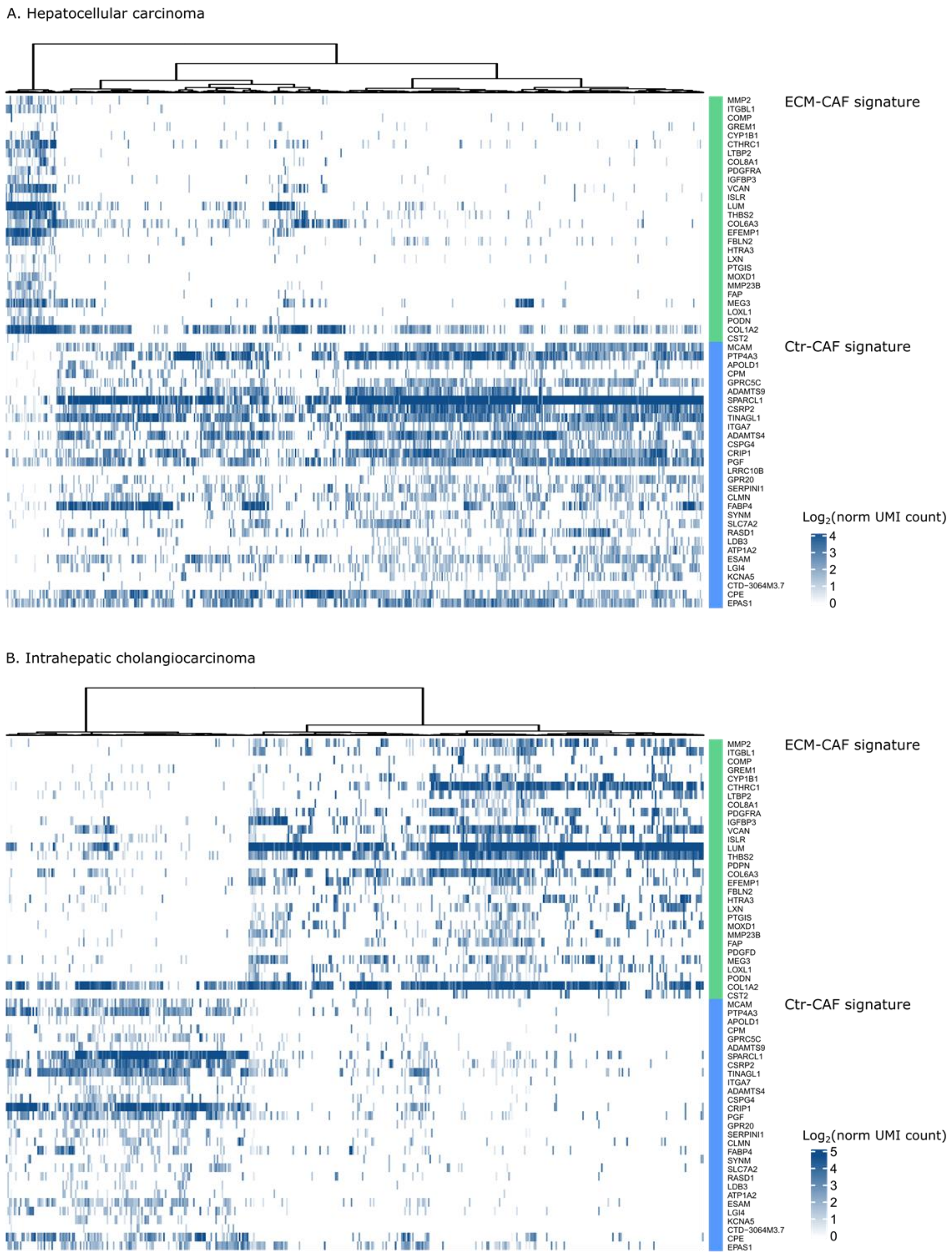

Supplementary Figure 24. LTBP2+ and MCAM+ gene signatures in hepatocellular carcinoma (A) and intrahepatic cholangiocarcinoma (B). Data from Ref.[7]

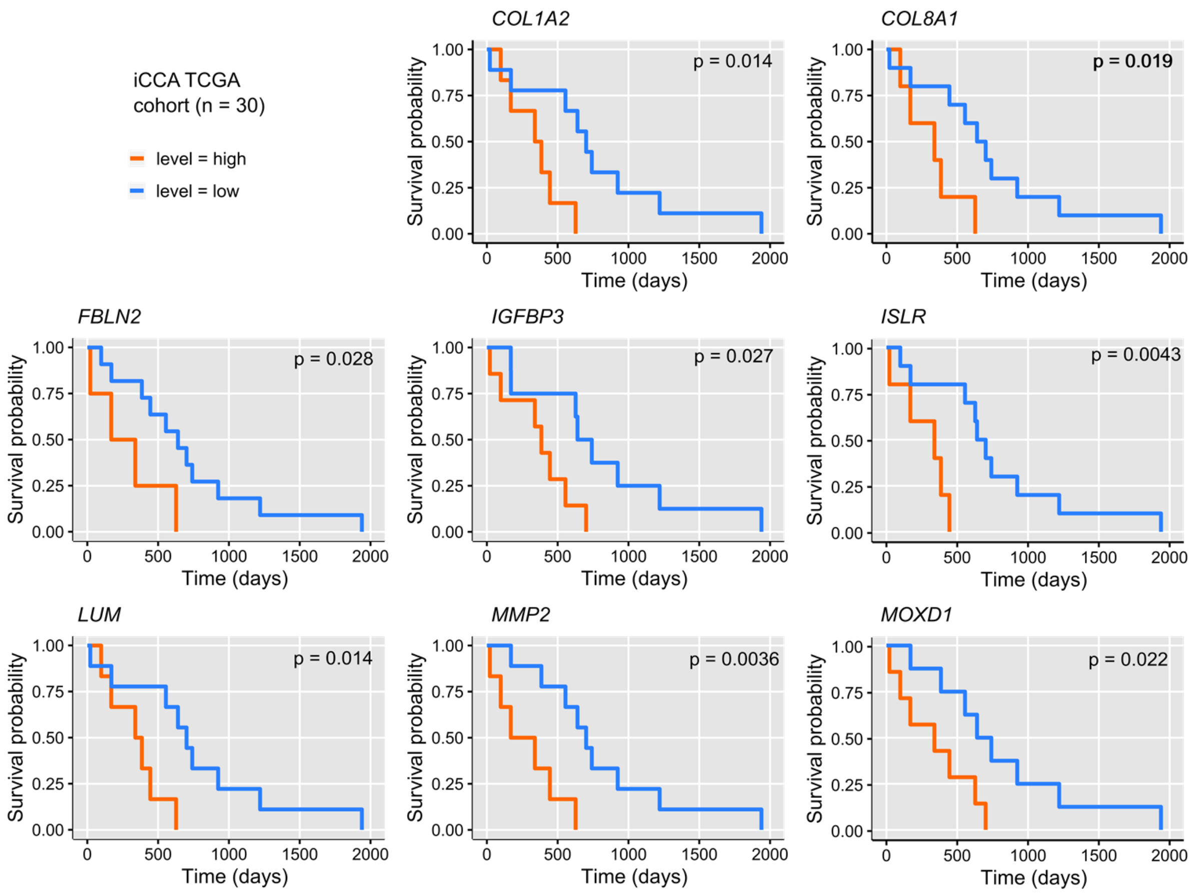

Supplementary Figure 25. Association between overall survival[8] and several genes of the ECM-CAF signature in TCGA intrahepatic CHOL (iCCA) patients. (Analyses were performed with R packages survival and survminer).

References

1 MacParland SA, Liu JC, Ma X-Z, Innes BT, Bartczak AM, Gage BK *et al.* Single cell RNA sequencing of human liver reveals distinct intrahepatic macrophage populations. *Nat Commun* 2018; **9**: 4383.

2 Gu Z, Eils R, Schlesner M. Complex heatmaps reveal patterns and correlations in multidimensional genomic data. *Bioinformatics* 2016; **32**: 2847–2849.

3 Qian J, Olbrecht S, Boeckx B, Vos H, Laoui D, Etlioglu E *et al.* A pan-cancer blueprint of the heterogeneous tumor microenvironment revealed by single-cell profiling. *Cell Res* 2020; **30**: 745–762.

4 Kieffer Y, Hocine HR, Gentric G, Pelon F, Bernard C, Bourachot B *et al.* Single-Cell Analysis Reveals Fibroblast Clusters Linked to Immunotherapy Resistance in Cancer. *Cancer Discov* 2020; **10**: 1330–1351.

5 Ramachandran P, Dobie R, Wilson-Kanamori JR, Dora EF, Henderson BEP, Luu NT *et al.* Resolving the fibrotic niche of human liver cirrhosis at single-cell level. *Nature* 2019; **575**: 512–518.

6 Che L-H, Liu J-W, Huo J-P, Luo R, Xu R-M, He C *et al.* A single-cell atlas of liver metastases of colorectal cancer reveals reprogramming of the tumor microenvironment in response to preoperative chemotherapy. *Cell Discov* 2021; **7**: 80.

7 Ma L, Hernandez MO, Zhao Y, Mehta M, Tran B, Kelly M *et al.* Tumor Cell Biodiversity Drives Microenvironmental Reprogramming in Liver Cancer. *Cancer Cell* 2019; **36**: 418-430.e6.
